# Ryūtō: network-flow based transcriptome reconstruction

**DOI:** 10.1186/s12859-019-2786-5

**Published:** 2019-04-16

**Authors:** Thomas Gatter, Peter F Stadler

**Affiliations:** 1 Bioinformatics Group, Department of Computer Science & Interdisciplinary Center for Bioinformatics, Universität Leipzig, Härtelstraße 16-18, Leipzig, 04107 Germany; 2 Max Planck Institute for Mathematics in the Sciences, Inselstraße 22, Leipzig, 04103 Germany; 3 Institute for Theoretical Chemistry, University of Vienna, Währingerstrasse 17, Wien, 1090 Austria; 4 Faculdad de Ciencias, Universidad Nacional de Colombia, Sede Bogotá, Ciudad Universitaria, Bogotá, D.C., COL-111321 Colombia; 5 Santa Fe Insitute, 1399 Hyde Park Rd., Santa Fe, NM 87501 USA

**Keywords:** RNA-seq, Transcript reconstruction, Splice graph, Exon bins, Bin graph, Minimum-cost flow

## Abstract

**Background:**

The rapid increase in High-throughput sequencing of RNA (RNA-seq) has led to tremendous improvements in the detection and reconstruction of both expressed coding and non-coding RNA transcripts. Yet, the complete and accurate annotation of the complex transcriptional output of not only the human genome has remained elusive. One of the critical bottlenecks in this endeavor is the computational reconstruction of transcript structures, due to high noise levels, technological limits, and other biases in the raw data.

**Results:**

We introduce several new and improved algorithms in a novel workflow for transcript assembly and quantification. We propose an extension of the common splice graph framework that combines aspects of overlap and bin graphs and makes it possible to efficiently use both multi-splice and paired-end information to the fullest extent. Phasing information of reads is used to further resolve loci. The decomposition of read coverage patterns is modeled as a minimum-cost flow problem to account for the unavoidable non-uniformities of RNA-seq data.

**Conclusion:**

Its performance compares favorably with state of the art methods on both simulated and real-life datasets. Ryūtō calls 1−4*%* more true transcripts, while calling 5−35*%* less false predictions compared to the next best competitor.

**Electronic supplementary material:**

The online version of this article (10.1186/s12859-019-2786-5) contains supplementary material, which is available to authorized users.

## Background

In recent years, high throughput sequencing has become the key to investigating the transcriptomes of both procaryotic and eukaryotic organisms [1, 2]. RNA-seq offers a high throughput, low cost methodology for direct sequencing of transcribed genes, thus for the first time enabling the effective identification and quantification of transcript isoforms. Compared to previous attempts to model genes de novo based on signals in the genome sequence in terms of coding regions and splice sites, RNA-seq offers a much more nuanced view of the actual structure of the products of transcription and processing. Still, the accurate classification of the transcriptional output remains a very challenging task, in particular for complex eukariotic genes. An increasing number of studies reveals the high degree of diversity in the transcriptomes of higher eukaryotes [3]. Genome-wide studies on human tissue revealed that 95% of protein coding multi-exon genes and 30% of non-coding RNAs undergo alternative splicing [4, 5]. Similar ratios have been found also for non-human targets, such as mice [6]. Complicating matters even further, existing annotations likely contain high error rates and are widely considered unreliable and incomplete. Analysis of large-scale RNA-seq experiments indicates that many rare isoforms have evaded annotation, and typically sized RNA-seq experiments miss out significant portions of low abundant spliceforms [7]. Yet, many analytic tasks rely on accurate and fast predictions of all transcripts as a basic first step of their pipelines, e.g. in gene regulation studies in embryonics [8] and diseases [1, 9, 10]. While the methods and algorithms described in this paper are also interesting for their own sake, improved predictions using our methods can therefore be useful for a number of tasks.

In a typical RNA-seq experiment, a set of up to 200 mio. paired-end reads is created, each between 75−150 basepairs (bp) in length. Assembling a set of short reads into a viable set of transcripts is subject to a series of challenges. Transcripts are known to have highly variable sequence coverage, even between isoforms of the same locus. As exons are commonly shared between isoforms, no unambiguous resolutions exist in many cases. In addition to such “natural” concerns, biases from subsequent processing steps or deriving out of the sequencing protocol have to be considered. As a well known example, both Ribo-Zero as well Poly-A selection will result in deviations in the read distribution [11], as well as other factors such as GC content or position [12, 13]. Therefore, the assumption of reasonably uniform coverage along single isoforms is generally violated. Methods have to account for and correct such errors whenever possible. Missing and misleading evidence routinely leads to false predictions even if all exons have been correctly identified [14]. Even if the transcripts are known, quantification remains difficult. Exon sharing and ambiguous read assignments, e.g. because of close paralogs, and low coverage are all known to limit quantification [15].

While an increasing number of methods have been published to solve both the transcript identification as well as the expression quantification problem [16–23], none of these existing approaches are truly satisfactory in terms of robustness, speed and at the same time accuracy of the results. While the achievable level of correctness is ultimately limited by various factors, most of all by noisy and incomplete base data, we propose several new and improved algorithms that significantly boost both quality and efficiency of transcript predictions. Our tool Ryūtō — named after spirit fires that appear as signs of the workings of water gods based in Japanese folklore, thus denoting our use of networks-flows — was designed as a general framework for transcript assembly with the expressed goal of later extension beyond the functional capability of current tools. In this first paper, we layout the foundation of this work. We show that Ryūtō identifies around 1−4*%* more true transcripts, while calling 5−35*%* less false predictions compared to the next best competitor Scallop [16]. We demonstrate improvements on both simulated as well as real-life datasets.

## Methods

### Overview

Ryūtō employs an extension of common splice graphs in combination with min-cost network-flows similar to Traph [19], as well as graph editing techniques related to Scallop [16] to improve results. Among many improvements in methodology, a key advantage is the ability to identify likely areas of errors in the assembly process as a starting point for rationally designing post-processing procedures. As a second, future, advantage, our implementation allows for straightforward integration of non-co-linear transcript or trans-splicing events.

Transcript assembly methods follow one of two general strategies. If a reference is available, the RNA-seq reads are aligned against the reference by a specialized state-of-the-art split alignment tools. Common choices include TopHat2 [24], STAR [25] or HISAT [26]. If no reference can be used or the reference is incomplete, a de novo approach can be chosen, where reads are directly assembled into transcripts. Although alignment tools also struggle to correctly assign reads due to e.g. similar regions in multi-copy gene families, sequencing errors or repeats, these issues are aggravated in de novo assemblies. Pure de novo methods therefore are usually less accurate and computationally more complex. Thus they are avoided wherever possible [27].

Ryūtō is set within a reference-driven framework, but also incorporates de novo concepts. In particular, Ryūtō can be used on mixed sets of inputs. Pertea et al. [17] first introduced a pipeline that assembled RNA-seq data into contigs that are then aligned against the same reference. Mixing contig alignments and conventional split read alignments can improve predictions. Due to the advanced use of long reads, Ryūtō can make effective use of such data.

Mapping split-reads against a reference results in a set of intervals within which reads provide evidence for (partial) segments of one or more exons. Split reads, i.e. splice junctions, indicate introns. Cufflinks [18], the most widely used transcript assembler, employs an overlap graph to consolidate this data. Here each fragment is represented by an individual node and nodes are connected if reads overlap and are compatible in their splice signals. This structure does well in conserving both evidences from reads spanning more than two exons, as well as paired-end connections, two commonly underused sources of information. The average exon length of many eukaryotic organisms, including human and mouse is smaller than 200bp [28]. Thus, not only reads spanning 1 or 2 exons, but also reads covering >2 exons are abundant in many datasets. The latter category, which we will refer to as *multi-splice*, can be used to resolve ambiguities among alternative splicing events. However, as fragments with less splice evidence are given the same importance in the graph, incorrect transcripts may still be chosen in practice, depending on the details of the post-processing procedure.

Additional evidence to resolve alternative splice sites can be found in paired-end information. However, in an overlap graph, reads of the same pair cannot be represented in the same node due to their unknown insert context. Their introns, on the other hand, can be tested for compatibility. Thus, an edge is added only if the reads themselves as well as their “partners” agree. While removing some complexity, this additional condition fails to resolve many cases, as ambiguous junctions remain in the unsequenced region between partners.

Splice (or connectivity) graphs provide an alternative mathematical framework. Here, full or partial exons are the nodes, and edges represent either splices or neighboring partial exons. Although several extension have been proposed for this type of graph, it is typically employed in its basic definition. The implied coverage of each node and edge can be leveraged for transcript extraction in terms of a network flow problem. Traph uses a min-cost flow to denoise the raw graph that is subsequently decomposed into isoforms. While abundance alone acts as a good classifier here, evidence from multi-splice and paired-end reads are neglected. The use of so called bin graphs has therefore been proposed e.g. in [21, 22], where reads are abstracted as sets of (partial) exons. Reads with the same evidenced set are grouped into bins that are then used as the new nodes in the generalized splice graph. Scallop only relies on a basic splice graph. However, it keeps track of phasing paths from multi-splice and unambiguous paired reads to resolve them via Linear Programming (LP) optimization operating on the uncorrected coverage values.

Ryūtō combines aspects of overlap and bin graphs with the aim of maximizing the use of both multi-splice and paired-end information to the fullest extent. To this end, we utilize a novel bin graph where multi-splice information stays maximally intact, while each read can still be matched to a unique path in the graph. It therefore retains the desirable properties of overlap graphs and at the same time provides access to the coverage values that have been proven to be effective in choosing transcripts. Non-uniformities are resolved using a minimum-cost flow problem that satisfies a minimum-square optimization criterion similar to the approach proposed by Tomescu et al. [19]. Flow-conservation allows us to simplify the graph followed by a setup of LP optimization similar to but more general than Scallop.

### Identification of exons and bins

Similar to other reference based transcript assemblers, Ryūtō relies on the output of a specialized spliced-alignment algorithm. Exons are identified as consecutive stretches of mapped regions, with splices indicating introns. If the intron of one isoform starts or ends within the boundaries of the exon of another, this exon needs to be split at this position and handled as two parts.

**Fig. 1:**
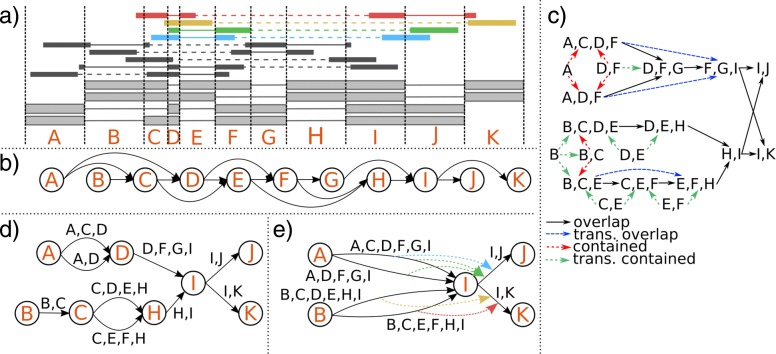
Overview of the most important steps of the transcript assembly pipeline. In (**a**) an example set of transcripts over the same gene is shown, together with potential paired-end reads leading to them. Reads have the same length *r*, but varying insert lengths. Be |*X*| the number of basepairs in exon *X*, then: |*A*|,|*C*|,|*D*|,|*E*|,|*F*|,|*G*|<*r* and |*C*|+|*D*|<*r*. All other conditions are >*r*. The commonly used split graph (**b**) removes all evidence from reads spanning more than two exons and paired-end information. Instead, an exon-bin based subset — adding edges between bins when a bin is a subset of another bin — and overlap — adding edges when a bin’s suffix is prefix of other bin — graph (**c**) is created (trivial subsets omitted). After removing transitive components, a directed acyclic splice graph is created that allows each bin to be uniquely mapped to a set of edges, but minimizes the number of ambiguous connections (**d**). Any maximum- or minimum-flow implementation can be used to establish transcript expressions on each edge. Due to flow conservation, composite paths and tree nodes can be reduced without loss of information. Flow decomposition into final transcripts can be improved by using evidences (**e**) from multi-exon-spanning single reads or paired-end data (dashed edge-links, color-code by (**a**))

We use 1D-clustering to resolve possible errors in alignments around junctions [29]. We first identify the smallest set of *n* exon ranges *X*=*x*_1_,…,*x*_*n*_, ordered by genome positions, that can explain all found splice-sites (Fig. 1a). We define a bin to be an ordered set of exons. Every read is assigned to a bin corresponding to all overlapping exon ranges in genomic order. Two partnered paired-end reads $r_{1} = x_{r_{1}^{1}}, \ldots x_{r_{1}^{i}}$ and $r_{2} = x_{r_{2}^{1}}, \ldots x_{r_{2}^{j}}$, |*r*_1_|=*i*, |*r*_2_|=*j*, are treated as a single read *z*=*r*_1_∪*r*_2_ if $r_{1}^{i} \geq r_{2}^{1}$ or $r_{1}^{i} + 1= r_{2}^{1}$, i.e., if they overlap or are consecutive without an intervening gap. Otherwise, paired information between reads will be stored for later steps. As a special feature of Ryūtō, genomic start- and end-positions of every read are stored in each bin in a compressed format. We keep detailed information of a reads only for (usually small) connected regions until its paired partner is resolved and counted. Otherwise we store read information as combined coverage gains and losses per base in a bin. As normally only a small portion of the genome is covered, with even fewer changes in coverage, this structure is very sparse, yet allowing full access to coverage motives at each bin or later exon in the graph. This technique allows us to read in complete chromosomes with very small memory footprint in a single pass. This will greatly simplify the later inclusions of trans-splices and other distant splice events. Similarly, bins from several input files over the same chromosome can be effectively merged.

### Graph construction

Instead of relying on traditional splice graphs, we propose a novel generalized bin-based graph structure that makes it possible to utilize multi-splice evidences. The nodes of *G* are identified such that they can represent a minimal partial set of exons *X* together with two artificial unlabeled nodes *s*,*t*∈*V* representing joined start and end points of all transcripts. Nodes *v*∈*V*(*G*)∖{*s*,*t*} are accordingly labeled *l*(*v*)∈*X* such that *l*(*v*)=*l*(*w*) implies *v*=*w*. The edges *e*∈*E*(*G*) correspond to (partial) bins and are labeled by ordered sets of exons *l*(*e*)={*l*_1_,*l*_2_,…} with *l*_*j*_∈*X*. The labels of *G* satisfy the following four conditions:

(i) Every path *p*=(*v*_1_,…,*v*_*k*_) such that *v*_1_=*s* and *v*_*k*_=*t* and *v*_*i*_*v*_*i*+1_∈*E*(*G*) for 1≤*i*<*k* through *G* corresponds to a unique transcript defined as the union $\bigcup _{e\in p} l(e)$.
(ii) Every bin *b*=(*x*_1_,…*x*_*j*_) maps to a unique path *p*=(*v*_1_,…,*v*_*k*_) such that *v*_*i*_*v*_*i*+1_∈*E*(*G*) for all 1≤*i*<*k*, *l*(*v*_1_)=*x*_1_, and *l*(*v*_*k*_)=*x*_*j*_.
(iii) *G* contains the minimal possible number of nodes among all graphs satisfying (i) and (ii).
(iv) For every edge *e*∈*E* the length |*l*(*e*)| is maximal.

Conditions (i) and (ii) hold for the basic splice graph (see Additional file 1: Note 1) but they are not necessarily satisfied for all bin graphs — e.g. StringTie [17] adds edges such that multi-splice bins create alternative paths signifying the same transcript for flow computations, violating both (i) and (ii). Since we enforce an injective but not necessarily surjective map from nodes to exons, condition (iii) maximizes exons that are only part of (possibly multiple) edges. Condition (iv) is required because edges can include exons that are also present as nodes (see following example). We note that our requirements to allow only one node per exon, as well as the sub-condition in (ii) requiring start and end exons of every bin to match nodes may seem restrictive at first glance. Indeed, it is easy to construct scenarios where we loose multi-splice information because of this definition. However, as bins tend to be incomplete for realistic data, both conditions have proven to help amend for such noise. Further, we exclude bins that are true subsets of a single unique bin from condition (ii). Similarly, we treat stretches of unique overlapping bins as a single bin for (ii). For a full discussion please see Additional file 1: Note 2.

The graph *G* can be computed in *O*(*N* log*N*) time if there are *N* initial bins.

We proceed in two steps to create our final graph. First we create an auxiliary graph $\phantom {\dot {i}\!}G^{\prime }=(V^{\prime },E^{\prime })$ that is used as a guide for the final bin graph. We initialize *V*^′^ as the set of all bins directly supported by reads. We add two kinds of edges $\phantom {\dot {i}\!}v^{\prime }w^{\prime }$, labeled accordingly (a) as *overlap* if the suffix of the bin *v*^′^ is a prefix of bin *w*^′^ or (b) *contained* if *v*^′^ is a subset of *w*^′^. This formulation is similar to the overlap graphs used by Cufflinks [18]. It uses an immediate bin-formulation, however. Using pre-sorted bins, this raw graph can be built in *O*(*N* log*N*) (see Additional file 1: Algorithm 1). *Contained* bins are not allowed to own *overlap* edges, which are removed accordingly. Both transitive *contained* and *overlap* edges are removed by using an adaptation of Myer’s approach for string graphs [30] in linear time. Pairs of nodes that are connected by a unique *overlap* path not overlapping to that of any other pair are successively merged. This is achieved in also linear time and in particular removes all nodes with in-degree 1 or out-degree 1. Thus merged bins are treated as a single bin according to (ii).

The resulting graph gives full evidence for creating the bin graph according to (i-iv) (see Additional file 1: Note 2).

We then use the following algorithm to build up the bin graph (see also Additional file 1: Algorithms 2 and 3): (a) Loop through all bins marked with *overlap* edges in the order of genomic position and create nodes for the first *v*_*i*_ and last *v*_*j*_ exons in the bin if they do not already exist in *G*. If there are no incoming *overlap* edges in *G*^′^, add the full bin as an edge *v*_*i*_*v*_*j*_ in *G*. Otherwise, split all paths in *G* corresponding to incoming *overlap* edges in *G*^′^ at position *v*_*i*_ and join them along the path to *v*_*j*_ to the rightmost pre-existing node *v*_*x*_<*v*_*j*_. Then add an edge *v*_*x*_*v*_*j*_ together with the corresponding partial bin. (b) Loop through all bins marked with outgoing *contained* edges in *G*^′^. If only one *contained* is marked, add the bin as a partial count to the path in *G* corresponding to the bin it is contained in (as exception to (ii)). Otherwise, join all paths corresponding to the containing bins in *G* along the bin.

The correctness of this procedure can be argued as follows. We reduced the auxiliary graph such that any *overlap* marks joints between contradictory bins. Hence, it is unclear to which bins the overlapping regions belong, and the paths in this regions need to be joined. Similarly, if a bin is contained in two or more longer bins, it is unclear to which one it belongs and paths needs to be joined as well. Therefore, we join exactly all regions conflicting with (ii) to achieve minimality according to (iii) and (iv) — see Additional file 1: Note 2 for a full discussion.

**Fig. 2:**
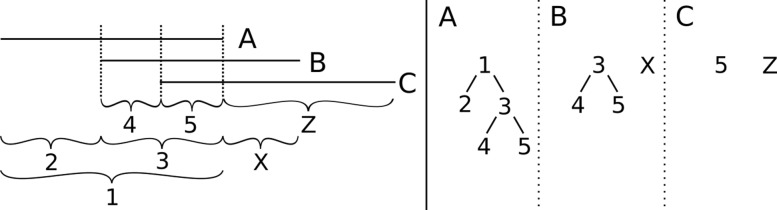
Shared interval markers are used to keep track of overlapping regions during computations. Each bin is assigned a directed binary forest of intervals, where current edges in *G* are marked as leaves, and inner nodes mark steps in the partitioning. Two distinct suffixes of bin A are respectively prefixes of bins B and C. B and C differ outside of the prefixes. When adding bins into the graph A will be added first by genomic position, and a single edge is added 1. B splits edge 1 into two edges 2 and 3, set as new child nodes to 1 in A. 3 and the new arc x are added as new trees in B. C further splits A, and with this indirectly B. As we use the same reference 3 for both bins, splitting is trivial. The resulting range forests are shown to the right

Remarkably, despite circular dependencies, we are also able to construct the splice graph in only linear time by using recursive, shared interval markers for each bin (see Fig. 2). In worst case, we compute exactly the basic split graph. In practice, however, we regularly see improvements.

### An example for graph construction

An example for advanced graph construction can be found in Fig. 1. Using a regular splice graph (b) for a set of transcripts (a) results in a loss of information. Instead, the auxiliary graph (c) created by Ryūtō shows that bins form distinct clusters that can be kept intact. Accordingly, in the bin graph (d) no nodes for exons E and F are created, despite their presence in multiple transcripts, as their bins uniquely define their connections. Exon D appears in a critical region between two transcripts and needs to be set as a node, as the bin (*D*,*F*) could belong to either one and both transcripts need to be compacted to fulfill (ii). A third isoform uses also D, but no otherwise violating bins force its connection to the established node. Instead, it is kept within the edge according to (iv).

### Inclusion of super-reads

Ryūtō can be used in combination with de novo sequence assembly using the methods invented by Pertea et al. [17] for StringTie. We used the script provided by these authors aimed to assemble contigs containing both reads for each pair utilizing the MaSuRCA assembler [31]. The resulting super-reads were aligned against the chromosome using HISAT or STAR. We merged the paired-end and super-read alignments into one file for benchmarks. As super-reads are by construction significantly longer than regular reads, they are more likely to include multiple splice-sites, resulting in longer bins. Ryūtō thus can use them to obtain a more accurate generalized splice graph. As suggested in [17], they may also map more accurately to the genome and therefore improve predictions in otherwise noisy or uncovered regions.

### Flow network design

Our generalized framework allowed us to test multiple flow network designs. We define cov(*v*) to be the coverage of the labeled exon on node *v*∈*V*(*G*) and cov(*uv*) the coverage of the bin corresponding to edge *uv*∈*E*(*G*). In general, we treat both nodes and edges in our bin-splice graph as flow constraints using cov as capacity in the network. Therefore, in practice, we convert each node *v* into two nodes *v*_*in*_, *v*_*out*_ joined by an edge *v*_*in*_*v*_*out*_ of capacity cov(*v*). We will omit this transformation in definitions for simplicity.

From a theoretical point of view, a simple maximum flow algorithm seems to be the most efficient implementation: Assuming perfect uniformity among transcripts, finding a maximal flow will show coverage saturation of all bins, with unused capacity indicating missed start- or end- sites. However, as uniformity is generally violated, we found this method to be inaccurate. The start- and end-sites tend to be underrepresented due to coverage biases, forming bottlenecks in the flow network and thus leading to an overall underestimation of transcripts and the introduction of incorrect additional starts and ends. We found no reasonable metric to distinguish bias from real evidences and therefore did not further pursue this path.

Any flow metric on splice graphs needs to account for this lack of uniformity in its definition. We therefore deviate from maximum flows and use a formulation based on the minimum-cost flow problem (MCFP) instead. We extended the Unannotated Transcript Expression Cover (UTEC) problem introduced by Tomescu et al. [19] for the use on our generalized graph structure. Given cost functions *c*_*v*_(.) and *c*_*vu*_(.) for *v*∈*V* and *uv*∈*E*, respectively, we aim to find tuples *P* of paths from source *s* to sink *t* with estimated costs *e*(*P*) such that we minimize

$$ \begin{aligned} &\sum\limits_{v \in V} c_{v}\left(|\text{cov}({v}) - \sum\limits_{p\in P: v \in p}e(p)| \right) + \\ &\sum\limits_{v\rightarrow u \in E} c_{vu}\left(|\text{cov}({uv}) - \sum\limits_{p\in P: uv \in p} e(p)| \right). \end{aligned} $$

In other words, we aim to find a set of transcripts with minimal errors to the coverage of each feature. The solution of Tomescu et al. via a minimum cost network flow on an offset graph can be directly applied to our bin graphs, because all relevant mathematical properties are preserved. As a result we obtain an optimal flow, quantifying each bin in the graph. Using the Network Simplex Algorithm we achieve this in *O*(|*V*|^2^|*E*| log(|*V*|*C*)) time, where *C* is the maximal cost of any edge. Since we chose convex cost functions, in our implementation a pseudo-polynomial transformation is necessary (see [32] for details). However, we strongly limit the arc-numbers using heuristics to avoid “blow-ups” in computation time that are common for Traph. Empirically, we found that *c*_*v*_(*x*)=*x*^2^/cov(*v*) and *c*_*vu*_(*x*)=*x*^2^(|*l*(*uv*)|−1)/cov(*vu*) are adequate cost functions that outperform those suggested by Tomescu et al.

Even though we confirmed that this method performs well on its own, we developed an additional, data driven, pre-processing step to denoise coverage labels. Flow formulations tends favor shorter transcripts that are often present in the graph due to incorrect alignments on splice sites as changing a single edge is often cheaper than changing a whole path. Even though the convex cost function aims to mitigate this influence, it does not completely remove the effect. We therefore pre-process coverage values to enhance uniformity along long transcripts first.

For each node, we compute the forward and reverse overhead: $b_{v}^{+}$ is the difference of the maximal coverage of *v* and the coverage of the rightmost base in *v*, and $b_{v}^{-}$ is the difference of the maximal coverage of *v* and the coverage of leftmost base in *v*, respectively. Then, we compute the forward correction of the coverage by updating nodes in topological order in the following manner:

$$\begin{array}{@{}rcl@{}} \text{cov}_{f}({v}) &=& \sum\limits_{u:u v \in E} (\text{cov}_{f}({u}) + b_{u}^{+}) \frac{\text{cov}({uv})}{\sum\limits_{z:z v \in E} \text{cov}({zv})} - b_{v}^{-}\\ \text{cov}_{f}({vu}) &=& \text{cov}_{f}({v}) \frac{\text{cov}({vu})}{\sum\limits_{z:v z \in E} \text{cov}({vz})} \end{array} $$

The reverse correction cov_*r*_(.) is analogously computed in reverse topological. We update coverage as cov(*x*)←cov(*x*)+ max(cov_*f*_(*x*),cov_*r*_(*x*)). These corrections, while related to the use of gain and loss factors for flow computations in combination with length restrictions in path selection in StringTie, are unique to Ryūtō.

If an annotation of known transcripts (guides) is available, we can make use of them to increase the accuracy of the denoising step. To this end we identify the paths corresponding to each guide, or remove the guide if it is incompatible with the graph. We compute the bias factor for each exon node as $b_{v} = \sum \limits _{u:v \rightarrow u \in E} \text {cov}({vu}) / \sum \limits _{u:u \rightarrow v \in E} \text {cov}({uv})$. We then determine the largest possible coverage for each transcript such that no coverage is exceeded on any edge or node, and coverage follows the bias exactly at each node by multiplying the factors along the path. As a result we obtain coverage values for each edge and node. We sum up values over all guides and compute the percentage *F* of coverage that that is accounted for in this manner. If the percentage exceeds a threshold defined by the user, we set the new coverage to the sum of guides; otherwise the coverage is increased to match *F*. This methods allows us to give the user a handle to account for particularly good or bad guides.

### Identification and quantification of transcripts

In order to extract transcripts as paths out of the flow network, we can make use of several well established properties of flows. A decomposition of a flow into at most |*E*| paths always exists and can be computed efficiently. Any such decomposition is optimal with respect to UTEC on the established flow, but not necessarily biologically meaningful. To explain transcipts *parsimouniously*, a minimal set of paths is usually sought. This is an NP-hard problem for which several alternative heuristics are in use [33, 34]. Most commonly, the heaviest paths, with maximal coverage, are removed successively until no flow remains. Traph, for examples uses this approach.

We observed that the minimal set of paths may not be the best metric, however. Our methods allowed us to solve the NP-problem for a substantial percentage of loci, resulting in overall worse classification. Instead of focusing on the heaviest path, we found that successively removing the longest possible transcript together with the maximal flow along its path performs better. This approach is reminiscent of StringTie, where the longest path containing the exon with the highest coverage in the raw split graph is selected first.

Prior to extracting transcripts, several simplifications can be made to the graph *G*. As a consequence of the flow conservation on nodes, we can remove tree nodes (with in- or out-degree of 1 and higher respective opposite degree) and composite paths (nodes with in- and out-degree 1). As a result, all inner (exon) nodes of the graph will have at least two in-coming and out-coming edges respectively, and therefore represent unresolved positions in the transcript assembly. We output such nodes for post-processing, because they identify likely points of mismatches in the subsequent assembly, as a unique feature of our method. This reduction in graph size also makes it possible to enumerate all paths locally left and right of each unresolved node. We employ a strategy reminiscent of Scallop, were the evidence is decomposed using Linear Programming (LP). Here we use a simple heuristic to mark transcript evidence: we mark all connections corresponding to overlapping (long) bins, or matching paired-end bins. However, we prioritize more specific hits, only adding less specific matches if more precise ones cannot sufficiently explain them. We chose this heuristic for several reasons. Foremost, in the presence of multi-splice bins, typically also bins exist that are subsets of these maximal bins, thus cannot provide new information and are removed. Additionally, we hope to exclude otherwise noisy bins. For example, if an exon *e*_*m*_ is shorter than the read size, but its flanking exons left *e*_*l*_ and right *e*_*r*_ are not, we can expect to see bins (*e*_*l*_,*e*_*m*_), (*e*_*m*_,*e*_*r*_), and (*e*_*l*_,*e*_*m*_,*e*_*r*_). Similarly, as the connection between read pairs is unknown, we might find connections between arcs in this gap that actually have incorrect labels. By prioritizing specificity, we hope to remove such errors.

We obtain a set of connections between incoming and outgoing edges. Given incoming edges *S*_*v*_ with evidence, and out-going *T*_*v*_ of a vertex *v* with connections *E*_*v*_:*S*_*v*_→*T*_*v*_, the number of reads *n*(.) inducing them, and the flow on each edge *fl*(.) we take two optimization steps using connection variables $\phantom {\dot {i}\!}x_{e,e^{\prime }} \in E_{v}$:

$$\begin{array}{@{}rcl@{}} \text{fl}(e) - \sum\limits_{e^{\prime} \in T_{v}: (e, e^{\prime})\in E_{v}} x_{e,e^{\prime}} &\leq & y_{e} \geq 0, \forall e \in S_{v}\\ \text{fl}(e) - \sum\limits_{e^{\prime} \in S_{v}: (e^{\prime}, e)\in E_{v}} x_{e^{\prime},e} &\leq & y_{e} \geq 0, \forall e \in T_{v} \end{array} $$

We first optimize subject to

$$\quad\text{minimize} \left| \sum\limits_{(e,e^{\prime}) \in E_{v}} \text{n}(e,e^{\prime}) - x_{e,e^{\prime}} \right| $$

to gain the minimal flow induced by each connection. We then take optimal $\phantom {\dot {i}\!}x_{e,e^{\prime }}$ as minimal values in a second step maximizing the used flow

$$\quad\text{minimize} \sum\limits_{e \in S_{v}} y_{e} + \sum\limits_{e \in T_{v}} y_{e} $$

Nodes with evidence are decomposed in the order of the quality of evidences and minimal square root error per flow. While conceptually similar to Scallop, our method has several advantages:

(1) We are not limited to pairs of reads with an unambiguous connection in the graph, but rather can include all pairs.

(2) Incomplete evidences will leave significant flow intact, where Scallop would remove edges.

(3) By prioritizing specificity, we create less false connections, and LPs are easier to solve.

(4) We are not required to resolve unmatched edges by forcing a connecting them by some heuristic. Rather, we can rely on flow decomposition to determine leftover complexity.

### Heuristics for noise reduction

Ryūtō handles noise stringently in each step of the computation. While noise reduction mechanisms remain often under-reported in publications, they are an integral part of every pipeline. For an exhaustive list of available filters, we refer to the description of the commandline options of the Ryūtō manual.

We found that much of Scallop’s success is not only explained by its LP strategies, but rather can be attributed to its smart noise filters. We therefore designed our settings after the same model, adding both reported and unreported procedures. As an added bonus, this ensures our reported improvements can explicitly be traced back to our novel algorithms. Most notably, nodes are categorized by quality of evidences. Edges without evidence and flow below a certain score threshold are removed in between steps. Ryūtō removes edges without evidence with less than 30% flow compared to any another edge incident to a common vertex, or less than 75% if no evidences are found for a node.

As the only additional filter, next to flow denoising, errors emerging from intron-retention are filtered by Ryūtō. Ryūtō will remove all (possibly interrupted) introns unless there is significant coverage evidence for them. This gives a slight advantage for noisy data, but a disadvantage for perfect data compared to Scallop.

Similar to Cufflinks and StringTie, Ryūtō can employ a threshold to filter out low abundance transcripts. By default, this behavior is disabled, because removing edges without evidence by the above criteria already works well on its own. However, for guided regions only transcripts with sufficient abundance compared to the most abundant transcript are reported, depending on the trust level given. This is necessary as guided transcripts are removed from the graph first, which obstructs the standard step-wise denoising.

## Results

### Benchmarking

In order to compare Ryūtō to competing programs, we benchmarked all tools on a diverse collection of datasets. We only considered tools tested positively by Hayer et al. [35], as well as newer, freely available, competitors. We had to omit Traph [19] and Strawberry [22], because they could not be run in the available time on the benchmark dataset or produced errors. This leaves only Cufflinks (v2.2.1), StringTie (v1.3.3), Scallop (v0.10.3), as well as Transcomb (v.1.0) for testing.

In order to conclusively benchmark our tool we need to consider both simulated, as well as real datasets. Results of simulated data were evaluated with the scripts provided by [35], calling correct transcripts only if all splices match and the same number of exons was predicted. Additionally, we require the predicted strand to match. We chose the same metric for real datasets, only here using Cuffcompare of the Cufflinks Package [18].

While Ryūtō and other tools were designed with mainly multi-exon transcripts in mind, especially those underlying alternative splicing, also single exon transcripts can be called by all tools. As the latter are more prone to be effected by fine tuned filter settings – and often can be called outside of the core methods of each tool by merely detecting areas of coverage – we will consider both benchmarks in- and excluding them.

We chose the combination of recall *vs.* precision as a key statistic in evaluating all tools. Naturally, improving recall will result in worse precision for each individual tool. We aim to compare all tools at a good trade-off point for both statistics, as an average user would. StringTie, Cufflinks, and Ryūtō can be run with standard parameter. Scallop and Transcomb do not filter significantly in their presets. Accordingly, we set Scallop to a minimal transcript coverage of 4, as this best matches the other tools. Transcomb was set to a filter value of 4 as well, although it did not compete well at any setting with decent precision.

It should be noted that any attempts to continuously match filters is heavily problematic. While each tool provides a key option to filter transcripts, and thus allow the user to adjust results, they ultimately all relate to different properties despite superficial similarities. Therefore, parameters cannot be simply set to the same value to the same effect.

### Simulated datasets

At present there are a no real life datasets for which the complete collection of transcripts and their expression levels are known with high precision. Benchmarking of transcript assemblers thus has to resort to simulated datasets. To stay objective, we used the benchmark datasets from a previous, independent evaluation of reference based transcript assemblers [35]. In the systematic dataset (T1) 13,000 artificial genes with varying numbers of isoforms, all of the same coverage, were simulated without sequencing errors. In the ENSEMBL Perfect (EP) and ENSEMBL Realistic (ER) sets all genes of *Mus musculus* mm9 annotated in ENSEMBL were simulated, with no errors and realistic error margins, respectively. For technical details on the datasets we refer to [35]. For each set, 50 million paired-end directional reads were created. All tools were provided with the same input and no guiding annotation.

**Fig. 3:**
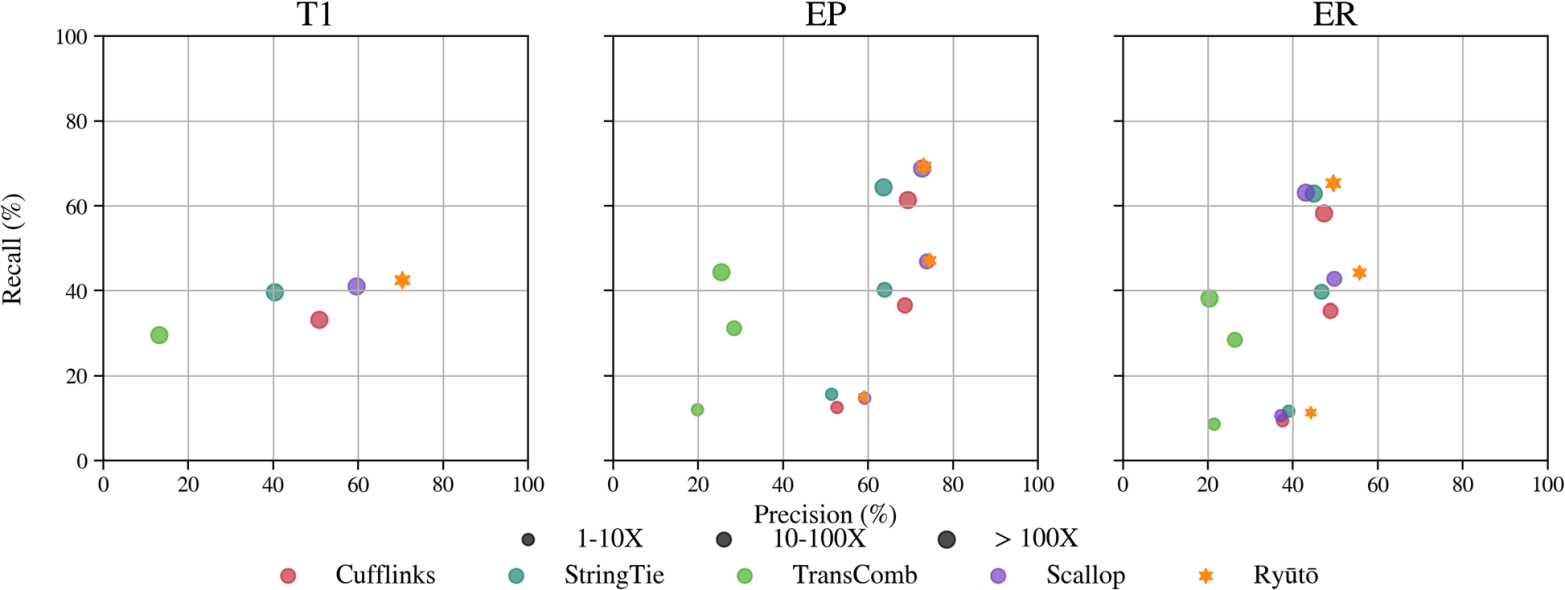
Accuracy of transcript assemblers as recall-precision plots summarized for the simulated datasets T1, ENSEMBL Perfect (EP) and ENSEMBL Realistic (ER) using the alignments produced with STAR. Plots are broken down by coverage of isoforms

Ryūtō performed significantly better on all datasets (Fig. 3; Additional file 1: Figure S10) in terms of the quality of the inferred isoforms. Ryūtō’s running times were comparable to StringTie and Scallop (see Additional file 1: Table S2).

**Fig. 4:**
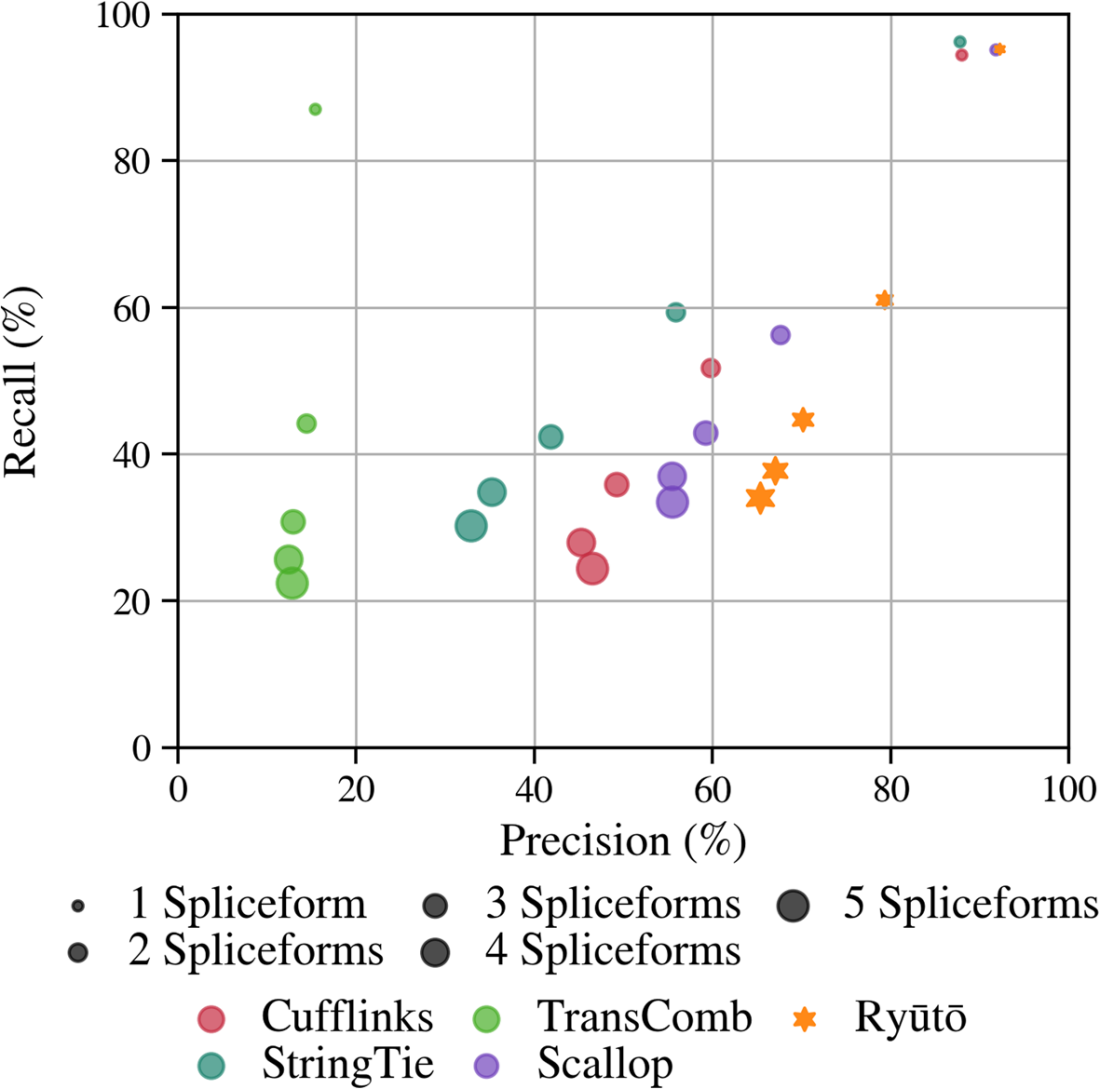
The artifical dataset T1 allows to break down accuracy (as recall-precision plots) along the number of spliceforms on genes. Ryūtō dominates in particular on genes with high splice variation

For dataset T1, Ryūtō found 3.6*%* more true transcripts compared to the next best performing tool Scallop, while producing more than 35.7*%* fewer false positives. Even for realistic data (ER) we still found nearly 3.6*%* more true transcripts and at same time reduced the false positives by 19.2*%*. This improvement is driven mainly by average to high abundant transcripts. For very low abundant isoforms Ryūtō performs very similar to StringTie and Scallop. Low abundant regions exhibit generally high levels of noise that can often not be easily distinguished from real data. Therefore, conservative filtering is employed as a preset among all tools with the exception of Transcomb. The artificial design of dataset T1 enables us to evaluate the impact of the number of spliceforms per locus (Fig. 4). While improvements can be seen in all categories, they are particularly prominent for more complex transcripts. Improvements are consistently observed for all alignment methods (Additional file 1: Figures S10 and S11). Ryūtō produces a significant number variants not called by other tools, both for true (see Additional file 1: Figures S12, S14, S16 and S18), but especially for falsely predicted isoforms (see Additional file 1: Figures S13, S15, S17 and S19)

With the exception of Ryūtō and Scallop, transcripts are mainly filtered in post-processing. Therefore, filters can only have a neutral or negative effect on recall. To complicate matters, filters vary wildly between tools, despite superficial similarities. Meaningfully matching similar options is difficult at best. As Ryūtō dominates in both categories for the overwhelming number of data-points, we did not systematically vary filter settings.

**Fig. 5:**
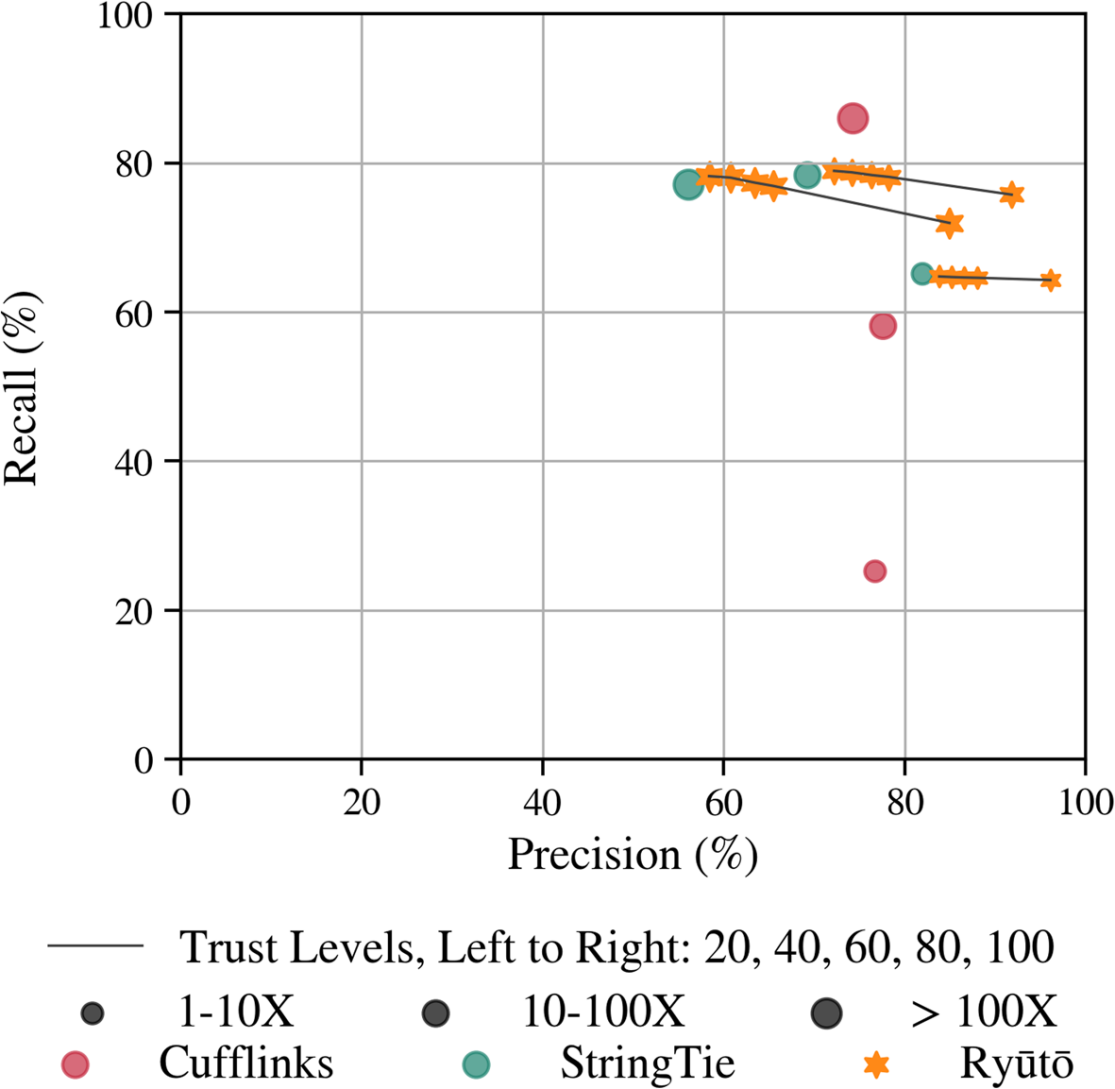
Ryūtō offers a trust parameter for guided transcript assembly as a unique feature, allowing the user to specify a trade-off between recall and precision

If gene coordinates are available to guide transcript assembly, they can be used to enhance predictions. In order to incorporate different levels of reliability we developed an abstract trust measure that gauges how reliable an annotation is perceived to be by the user. Ideally, unannotated transcripts will only be called if the annotation can not sufficiently explain the data, and increasing trust levels should decrease the number of unannotated transcripts while increasing precision. We had to exclude Transcomb and Scallop from this test, as they provide no guide option. To simulate unreliable annotations, Hayer et al. [35] provides guides where 15% of transcripts were removed and replaced by unexpressed isoforms. Figure 5 summarized the performance of different transcript assemblers.

While Cufflinks showed impressive accuracy for highly abundant transcripts, recall for others was much worse. At medium trust levels, we found that Ryūtō improves medium to high abundant recall, while performing only comparable in very low abundant ones. We have yet to investigate this discrepancy in detail, but found that StringTie e.g. calls annotated isoforms even though individual splices have not been observed, but exons are still present. We have not yet implemented such a system. Most noteworthy, for the highest trust rating 100, we only loose 3.1*%* of true transcripts compared to StringTie (2.9*%* to trust 60), compared to 80.5*%* (72.3*%* to trust 60) fewer false positive predictions, despite the moderate annotation quality.

### Isoform expression

Network flow approaches closely link the quantification of the expression levels of individual isoforms with the isoform identification. In order to benchmark the inference of (relative) expression levels, we computed the Spearman’s rank correlation coefficient to the ground truth for each individual chromosome. To this end, FPKM (fragments per kilobase of transcript per million fragments) values are first converted to ranks in increasing order that are then correlated to the true ranks. To avoid the problem of assigning ranks to 0 values (from false positive or unpredicted transcripts), we restrict the evaluation to true transcripts. This performance measure favors tools with low recall but avoids biases from lower precision and thus somewhat handicaps our own tool. Nevertheless, we found that correlation is on par or improved. Cufflinks, with substantially smaller isoform recall, achieves only slightly better scores (see Additional file 1: Table S3). Since Scallop explicitly recommends the use of a second tool for quantification, while internally relying on abstract scores, it was not considered in this test.

As there is no way of knowing true abundances of real datasets, we could only compute this measure for the simulated datasets. T1 was designed with equal abundances for each transcript, therefore offering no rank order. As expected, all tools show *ρ*≈0 reflecting this fact.

### Inclusion of de novo reads

As mentioned above, Ryūtō can be run utilizing also de novo concepts. We assembled the paired reads of ER to super-reads according to the StringTie pipeline and aligned them against the reference genome using HISAT and STAR aligner. Transcomb was unable to run on merged alignments, and thus not considered for this task. Cufflink’s accuracy is well documented to detoriate for this use-case in [17], hence it was also not considered here.

**Fig. 6:**
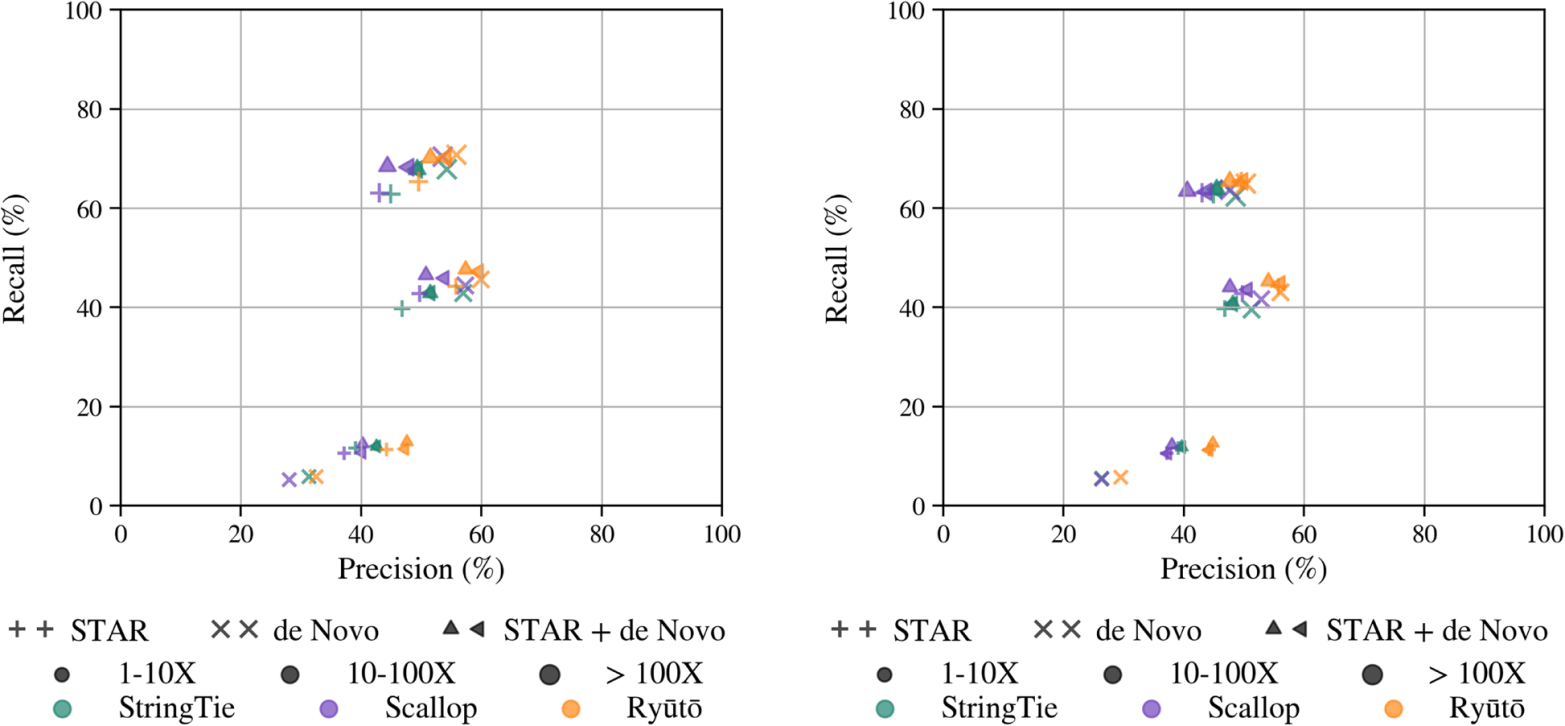
Ryūtō, Scallop, and StringTie allow the use of aligned de novo assembled super-reads next to normal paired-end alignments, all showing improvements when using this method, both using the more accurate HISAT (l.h.s.) or to a lesser degree STAR (r.h.s.) for de novo alignment. Combined alignments were run in standard setting (up-pointing triangle) as well as with filter coverage 5 (left-pointing triangle)

StringTie, Scallop, and Ryūtō all showed improvements (Fig. 6). We ran all tools both in standard setting, as well as with slightly increased coverage filters to offset the increase in input size. As HISAT alignments are more accurate, the observed gains were also larger. For STAR, true improvements were only reached using the second option, as otherwise too much additional noise was kept. Changes were consistent among all tools, relative to the the respective base values. It is worth noting that results for HISAT de novo alignments alone were consistently more accurate compared to paired-end alone for all but low abundant isoforms.

### Real datasets

Even though RNA-seq simulators strife to capture the variety of real data, they might fail to reproduce some aspects. Yet, when testing on real data it is impossible to know which genes or isoforms are expressed nor their expression levels. Nevertheless, we have curated sets of known genes for well studied organisms, including human and mice, that can be used as a reasonable approximations of the truth. In particular, it is fairly safe to assume that a prediction that matches a curated gene is most likely true. In the following we also make the assumption that all other predictions are false positives. This is not necessarily true – they may just have remained unannotated so far. As all tools are penalized equally, we suppose that these caveats have no crucial influence on the ranking, only on the distance between tools. We used eight datasets of human, and two datasets from mouse ranging from 25 to 167 Mio. spots utilizing 76 or 101 Bp paired-end, stranded protocols. To avoid any bias, Datasets were mainly chosen from publications of competing tools while representing a broad range of sample sizes and read lengths (see Table 1).

**Table 1:** Summary of RNA-seq samples used in this paper

Organism	SRA Accession	GEO Accession	Chosen By	*#* Spots	Cell Line	Localization	Length
human	SRR307911	GSM758566	TransComb, Scallop	41M	H1-hESC	cell	76
	SRR307912	GSM758566	-	36M	H1-hESC	cell	76
	SRR387661	GSM840137	TransComb, Scallop	125M	K562	cytosol	76
	SRR307903	GSM758562	Scallop	36M	BJ	cell	76
	SRR534319	GSM981256	StringTie, Scallop	25M	CD20+	cell	76
	SRR545695	GSM984609	StringTie	40M	CD14+	cell	76
	SRR534307	GSM981252	Scallop	167M	MCF-7	cytosol	101
	SRR545723	GSM984621	Scallop	147M	HMEpC	cell	101
mouse	SRR203276	SRX062280	TransComb	52M	dendritic	cell	76
	ERR1138641	ERX1217510	-	29M	liver	cell	101

**Fig. 7:**
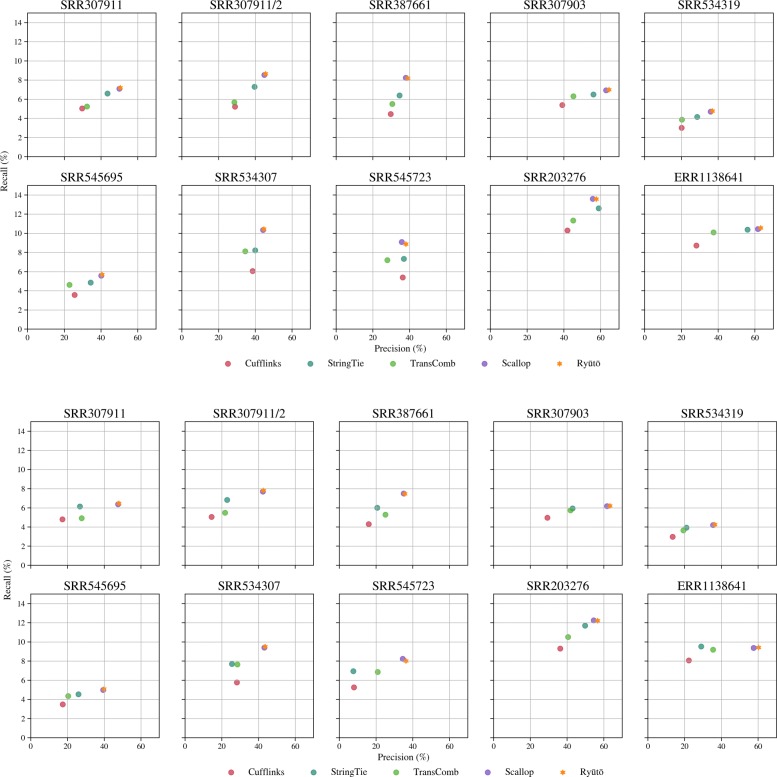
The advantage of Ryūtō also becomes apparent in real datasets downloaded from NCBI, again using recall-precision plots, both only comparing multi-exon transcipts (top), or all transcripts (bottom)

Improvements on calling only multi-exon were consistent and systematic among all datasets (Fig. 7), with Ryūtō calling more true transcripts (up to 2%), while calling significantly fewer false ones (up to 5.9*%*). As any improvement in recall entails a larger factor of false positives, Ryūtō significantly improves accuracy. When matching Scallop to the recall of Ryūtō, by slight variations in filter settings, we find that it calls up to 12.7*%* more false positives, averaging at around 5%. For SRR545723 Ryūtō filters stronger than Scallop, likely due the additional intron filter. Disabling this filter reveals a much closer match regarding recall while still outperforming Scallop’s accuracy (see Additional file 1: Table S11). Like for the simulated data, Ryūtō produces a significant number variants not called by other tools, both for true (see Additional file 1: Figures S12 and S16), but especially for falsely predicted isoforms (see Additional file 1: Figures S13 and S17).

Similar results were achieved when including also single-exon transcripts into the metrics (Fig. 7, Additional file 1: Table S12), although Ryūtō’s advantage in accurate compared Scallop decreases by a minor factor. Again, Ryūtō produces a significant number of variants not called by other tools (see Additional file 1: Figures S14, S15 S18 and S19).

## Conclusion

Ryūtō uses aspects of overlap graphs to create a generalized splice graph to make use of multi-splice evidences. A network-flow is used to assemble and quantify reads. Local enumeration at problematic graph regions has made integration of paired-end data possible to resolve ambiguities. Compared to other leading methods, Ryūtō is significantly and consistently more accurate for both simulated and real data, in particular for complex loci. Ryūtō can also be used with de novo assembled super-reads that combine pairs of reads, providing an additional increase mostly among low and medium abundant loci. It includes a framework for guided transcript assembly that can help adjust predictions according to annotation quality and user preference as a completely unique feature.

The Ryūtō framework in its current implementation is already a competitive alternative for the task of isoform identification and isoform quantification in RNA-Seq pipelines that can achieve substantial improvements. By design, Ryūtō’s internal data structures lend themselves to handling circular and trans-splice events in future versions of the software. The same features make it possible to localize positions in the graph structure that are the likely cause for errors, a property that will be useful for future improvements. Its structure also facilitates an easy exchange of components, allowing for easy prototyping and evaluation of individual elements. We reserve a discussion of alternative components that can help guide further development for another paper, especially in regards to noise handling.

## Additional file

Additional file 1: Supplementary materials This file contains additional information for graph construction and supplementary figures and tables. (PDF 10, 402 KB)

## Abbreviations

EP: ENSEMBL perfect
ER: ENSEMBL realistic
Fig.: Figure
LP: Linear programming
T1: Systematic dataset
Tbl.: Table
