# Supplementary material for: Ryūtō: network-flow based transcriptome reconstruction
Source: BMC Bioinformatics. 2019 Apr 16;20:190. doi: 10.1186/s12859-019-2786-5 (PMC6469118; doi:10.1186/s12859-019-2786-5)
Supplement: Supplementary file 1 — This file contains additional information for graph construction and supplementary figures and tables. (PDF 10, 402 KB) [file 12859_2019_2786_MOESM1_ESM.pdf]

Supplementary Materials

Additional Implementation Details

Alignments

We tested results on alignments created by STAR [Dobin et al., 2013], TopHat [Kim et al., 2013] and perfect alignments. For thesimulated datasets we relied on the alignments provided by Hayer et al. [2015]. HISAT [Kim et al., 2015] proved to be more accurate for the simulated datasets, but could not be generally included as Transcomb is unable to use the resulting alignments. All others aligners will include the bam-flags required by Transcomb, although generally only TopHat is recommended by the creators. As results do not seem to be impacted strongly, we nevertheless use all options for the tool.

We used standard parameters for all alignments, however, set STAR to filter splices to contain only consensus splice motives.

For human data we used GRCh37/HG19 as the reference genome, with accordingly GENCODE v19 as the latest available annotation as base truth. For mouse we used NCBIM37/MM9 with GENCODE vM1.

Implementation

Ryūtō was built in C++ with the help of several libraries. We use the Lemon Graph Library [lem] for all graphs. LP instances are solved with CLP [clp]. Boost [boo] datastructures are used for efficiency. Lastly, we used HTSlib [hts] for handling alignments files.

Parallelization

We use OpenMP to parallelize computations when possible. In particular, we allow the user to process multiple chromosomes concurrently via a simple commandline parameter. As transcripts naturally form many independent relatively small graphs, these can also be treated individually and in parallel.

Notes

Supplementary Note 1: Proof that properties (i) and (ii) hold for splice graphs.

By design, a splice graph consists of (partial) exons as nodes, with edges representing either splices or neighboring partial exons. Each node is therefore assigned a unique genomic interval, and splices or connections between neighboring partial exons map to unique edges accordingly. Each splice of each bin is recorded. Each exon maps to exactly one node and vice versa.

Let us assume that two paths exist that differ in at least one edge but represent the same transcript, thus violating (i). Then, two different edges would indicate the same genomic feature, therefore contradicting the definition of the splice graph.

Similarly, let us assume that a bin maps to two paths that differ in at least on edge in the graph. Again, this would require for two edges to indicate the same genomic feature and contradict definitions. Assume a bin does not match any path, at least one splice of the bin was not recorded in the graph, contradicting its definition. As all exons are represented as nodes and edges can only contain single splices, bin starts and ends necessarily match nodes.

This completes the proof.

Supplementary Note 2: Discussion of graph creation according to (i)-(iv).

The design of bin graphs is a non-trivial tasks due to a multitude of restrictions and objectives. We consider three major design criteria: (a) Stability of the design given incomplete data. (b) Preservation of evidence from multi-splice bins. (c) Fast implementation. Objectives (a) and (b) in general contradict each other. In order to test this trade off, we modified our implementation to maximize (b) as follows: We now allow $l(v) = l(w)$ even if $v \neq w$, thus representing exons as multiple nodes as needed. Additionally, we

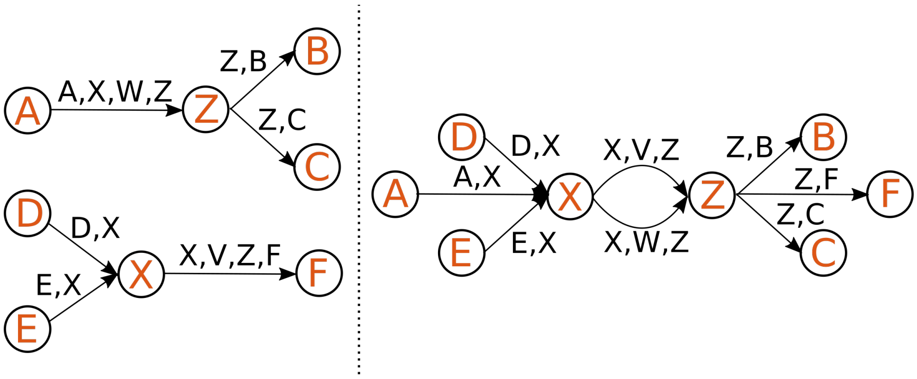

Figure S8: Information loss of the original definition (right) compared to the alternative definition (left). We have exon bins of unrealistic theoretical quality: AXWZ, XWZB, XWZC, DXVZ, EXVZ, and XVZF. The bins form two cluster in the overlap graph that stay intact in the alternative definition. As we normally require bins to end at nodes and nodes are unique per exon, both clusters are contracted into one graph component. We argue that this case is rare.

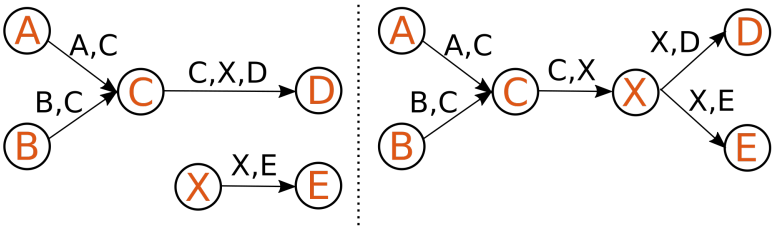

Figure S9: Information gain of the original definition (right) compared to the alternative definition (left). Exon X is smaller than the readsize. However, only bins AC, BC, CXD, and XE are present, with the expected bin CXE missing. Condition (iv) forces bin XE to stay disconnected for the alternative condition. In the original formulation, (ii) forces the presence of a node for X, and XE is joined as nodes are unique. This is a common problem, especially in low abundant regions.

drop the constraint that $l(v_1) = x_1$, and $l(v_k) = x_j$ from (ii). We can use a modified version of the original algorithm for this purpose, and leave details as an exercise for the reader. The thus enforced integrity of multi-splice bins has a positive impact in some instances (Suppl. Fig. S8), but shows bad properties on incomplete bin sets (Suppl. Fig. S9). Overall, on realistic data, the quality of description decreases (see Suppl. Tbl. S10).

In order to show the correctness and maximality according to (iii) and (iv) of our original algorithm, we argue by listing elemental operations on the overlap graph, corresponding to the resulting bin graph structures they induce. We can then depict the algorithm as a series of elemental operations, each contracting a group of nodes in the overlap graph into a single node until only a single node per component remains. Given the operations, a simple proof by induction can be formulated: As the bin graph is acyclic, the inverse of the listed operations can be used to build up any overlap graph. Local maximality is also true on a global perspective, as the restricting factors remain the same. In order to minimize visual elements, we here do not label edges with the nodes of a bin as before, but rather use placeholder variables that represent ≥ 1 splices each. Different naming strictly indicates incompatible splices, while same names represent the same splice signal.

ID	***Bins***	***Overlap-Configuration***	***Overlap Operation***	***Graph Operation***
2.1	a, b<br>ax, bx<br>xa, xb<br>a_1xa_2, b_1xb_2	(a)<br>(b)	(a)<br>(b)	○ —a→ ○<br>○ —b→ ○
2.2	ax, xb	(ax) → (xb)	(axb)	

2.3	a_1xa_2, x<br>ax, x<br>xa, x
3.1a	ax, bx, xc
3.1b	axy, bx, xyc
3.2a	xa, xb, cx
3.2b	yxa, xb, cyx
3.3	a_1xa_2, b_1xb_2, x
3.4a	ax, xb, x
3.4b	x, xy, xyb<br>x, xy, bxy<br>x, xy, b_1xyb_2
3.5	axy, xyz, yzb

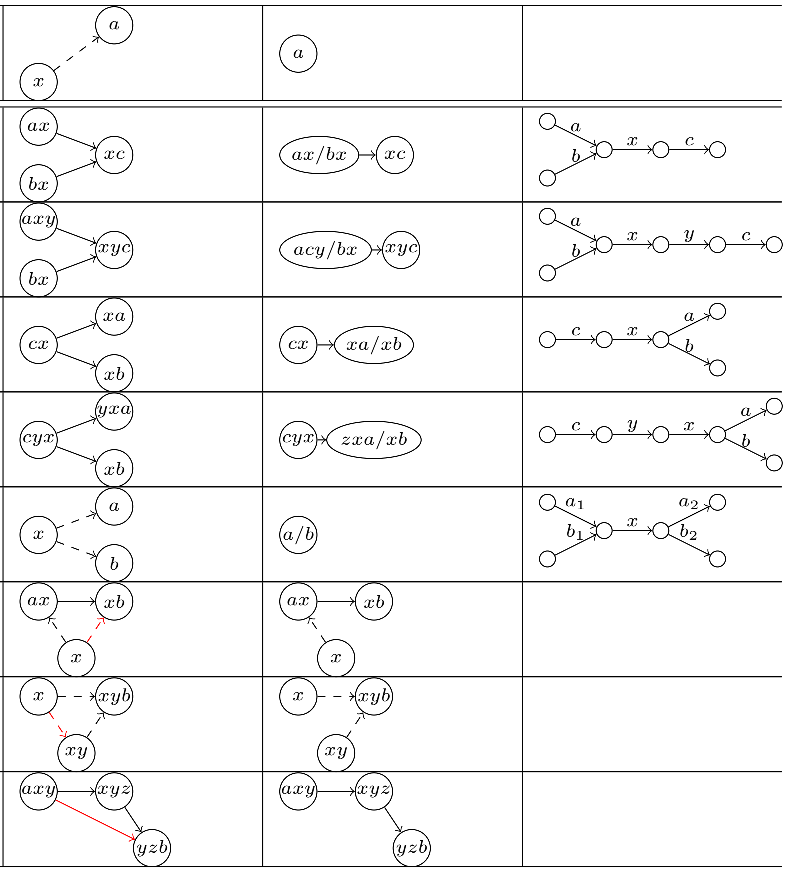

2.1 Bins have no overlap or containment relation. Whole inclusion of bins in edges does not contradict (i) or (ii), as bins are distinct. We therefore use the identity function of each component in the bin graph if it exists, or otherwise a single edge. Cases with more bins follow analogously. **2.2** We treat unique overlaps as a single bin. Therefore, inclusion of a middle node according to (ii) is not required and maximality forces no changes. **2.3** We disregard bins that are unique subsets of another bin. Therefore, no inclusion of middle nodes according to (ii) are required and maximality forces no changes. **3.1a/b** As a prefix overlaps to at least two nodes, unique mapping of bins (ii) is violated unless paths are joint at the largest overlapping suffix for each incoming node. According to (ii) every bin bin needs to end at a node, inducing nodes at every prefix corresponding to a suffix of an incoming node. **3.2a/b** As a suffix overlaps to at least two nodes, unique mapping of bins (ii) is violated unless paths are joint at the largest overlapping prefix for each outgoing node. According to (ii) every bin bin needs to start at a node, inducing nodes at every suffix corresponding to a prefix of an outgoing node. **3.3** As a bin is contained in two edges, it can not be uniquely mapped, violating (ii). Therefore, both need to be joined. **3.4a** A bin contained in the overlapping region of two nodes actually belongs to a single path. **3.4b** A bin contained in an also contained bin does not induce any violations to (i) or (ii). **3.5** Transitive overlaps can be ignored, as pairwise treatment induces the same data.

Please note that instances of 3.4a, 3.4b, 3.5 need to be resolved before the rest of all operations, with the exception of 2.2, which can only be used last per locus, as all more specific operators need to go first. Of course, induced bin graphs need to be preserved and updated for each step. Nodes cannot be lost and edges only get split, as they were mandated for by (i) or (ii).

Operations 3.4a, 3.4b, 3.5 correlate to the removal of transitive edges in our algorithm. 2.2 is applied to nodes with in-degree 1 or out-degree 1. 3.1 and 3.2 correspond to part (a) of the algorithm, 3.3 to (b).

We give an example decomposition as follows:

Operation	***Overlap Graph***	***Bin Graph***
	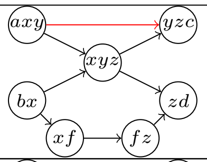	
3.5	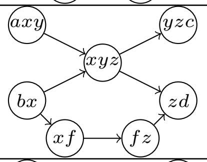	
2.2	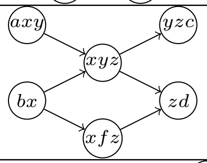	
3.1b	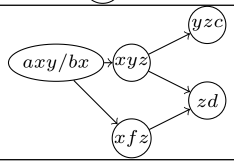	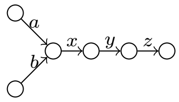
3.1a	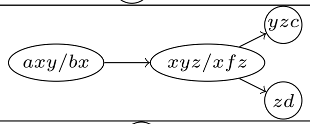	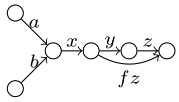
2.2	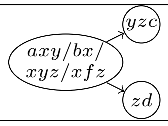	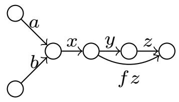
3.2b	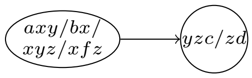	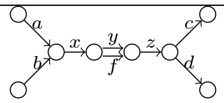
2.2	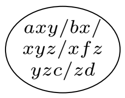	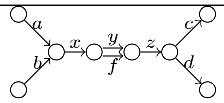

Algorithm 1 Generate the overlap graph from bins.

Require: list of bins B, empty graph G'
  sort B according to genomic position of each exon
  active bins $A := \{\}$
  contained $c :=$ **false**
  for $b \in B$ **do**
    for $a \in A$ **do**
      if genomic position of a left of leftmost exon in b **then**
        $A := A \setminus \{a\}$
      else
        if b subset of a **then**
          add *contained* edge ba in G'
          $c :=$ **true**
        end if
      end if
    end for
    if $c =$ **false then**
      for $a \in A$ **do**
        if suffix of b is prefix of a **then**
          add *overlap* edge ab in G'
        end if
      end for
      $A := A \cup \{b\}$
    end if
  end for
  reduce transitive edges on G' in post-processing

Algorithm 2 Generate the bin graph from the overlap graph. We omit details of range handling.

```
Require: overlap graph G′, B_n overlapping nodes, B_c contained nodes
  for b ∈ B_n in genomic order, |b| = k  do                                  ▷ (a)
    if b has ≥ 2 incoming overlap edges then
      c incoming overlap edge, range of overlapping bin i − j
      find edge e of c including i
      split e at i
      if  b_j not a node then
        create it
      end if
      add edge b_j b_k with label b_j . . . b_k to G
      for next c do
        for edges e_b of b do
          if c has a node in range of e_b at position i then
            split e_b at i
          else if e_b induces a node not in any edge e_c of c at position i then
            split e_c at i
          end if
        end for
      end for
    else
      if  b_0 not a node then
        create it
      end if
      if  b_k not a node then
        create it
      end if
      add edge b_0 b_k with label b to G
    end if
  end for
  for b ∈ B_c in genomic order, |b| = k, range of b bin i − j  do             ▷ (b)
    c outgoing contain edge,
    find edge e_i of c including i
    split e_i at i
    find edge e_j of c including j
    split e_j at j
    for next c do
      for edges e_b of b do
        if c has a node in range of e_b at position i then
          split e_b at i
        else if e_b induces a node not in any edge e_c of c at position i then
          split e_c at i
        end if
      end for
    end for
  end for
```

Algorithm 3 Split an edge in the bin graph for Alg. 2

Require: Graph G, Map m of edge labels in G, split position i, edge $e = uv$ to split with bin b, $|b| = j$

```
if b_i = u or i = v then
  return
end if
if b_i not a node then
  create it
end if
if b_0 ... b_i not in m then
  add edge b_0 b_i with label b_0 ... b_i to G
  add b_0 ... b_i to m
end if
if b_i ... b_j not in m then
  add edge b_i b_j with label b_i ... b_j to G
  add b_i ... b_j to m
end if
split range at i and assign edges
remove e from m
remove e from G
```

Additional Figures

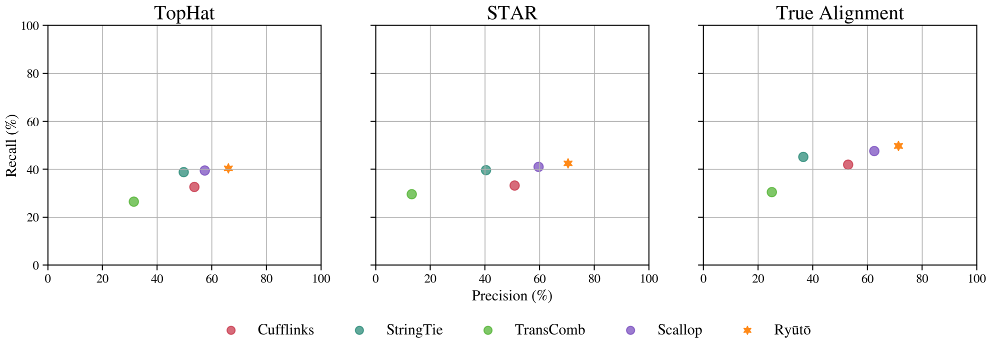

(a) All Alignments T1

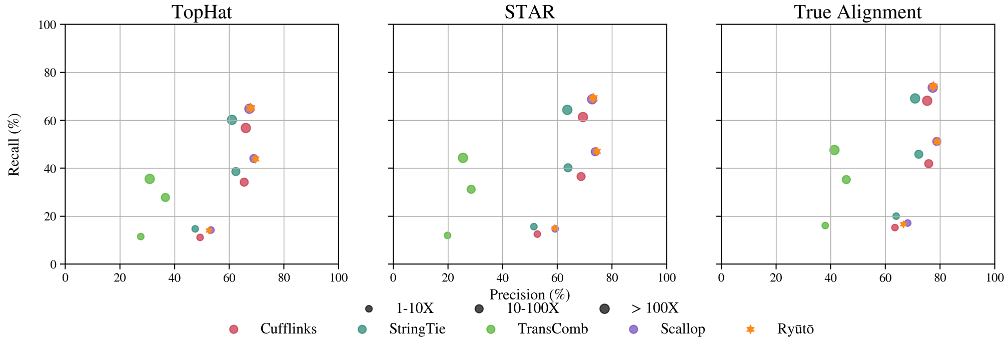

(b) All Alignments EP

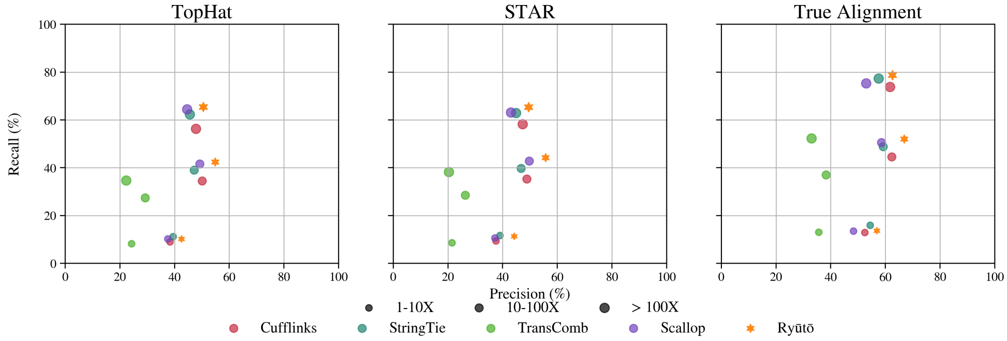

(c) All Alignments ER

Figure S10: Accuracy of transcript assembly for all tools and alignment methods for the simulated datasets (a) T1, (b) ENSEMBL Perfect and (c) ENSEMBL Realistic. Results are separated corresponding to low, middle and high abundant transcripts.

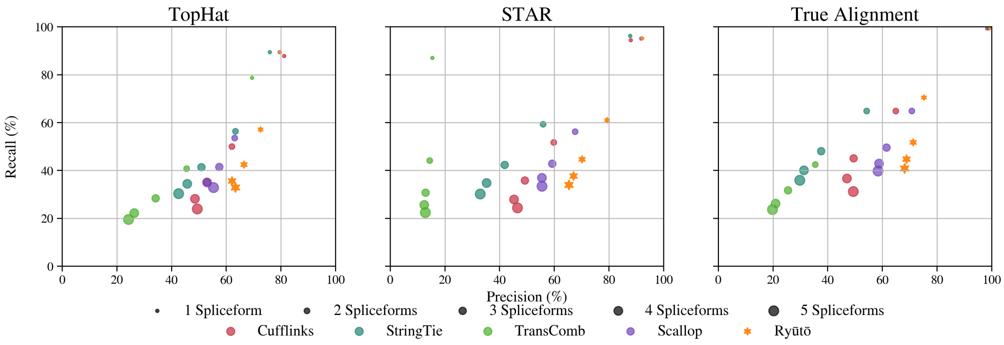

Figure S11: Accuracy of transcript assembly for all tools and alignment methods for the theoretical simulated dataset T1. Artificial genes have maximal 5 spliceforms. Results are broken down according to this number of spliceforms for each locus.

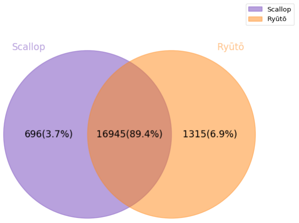

(a) T1 True Predictions

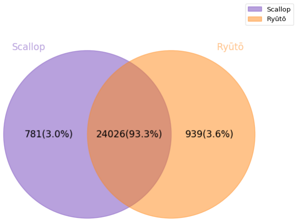

(b) EP True Predictions

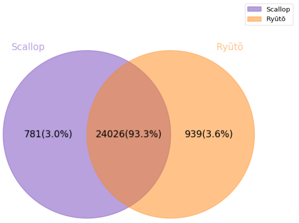

(c) ER True Predictions

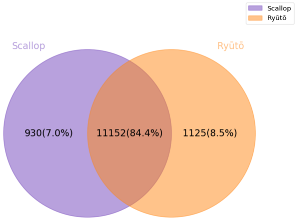

(d) SRR307911 True Predictions

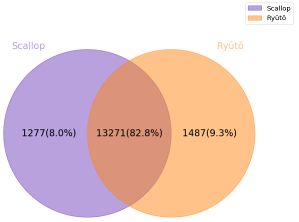

(e) SRR30791_1_2 True Predictions

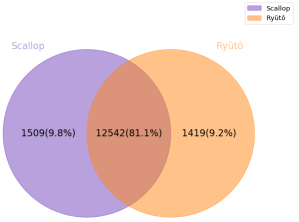

(f) SRR387661 True Predictions

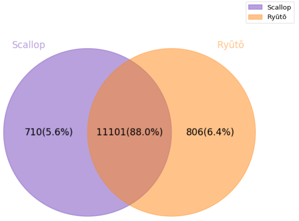

(g) SRR307903 True Predictions

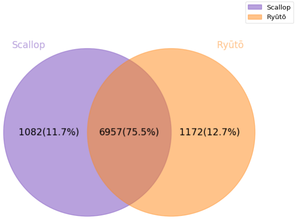

(h) SRR534319 True Predictions

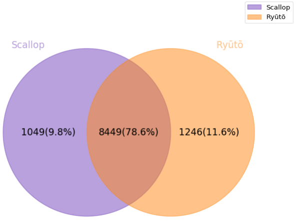

(i) SRR545695 True Predictions

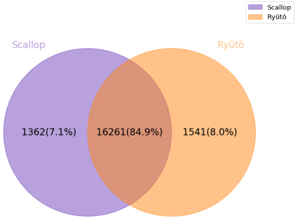

(j) SRR534307 True Predictions

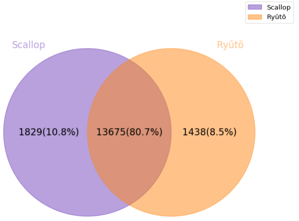

(k) SRR545723 True Predictions

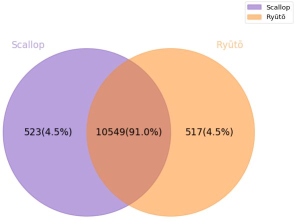

(l) SRR203276 True Predictions

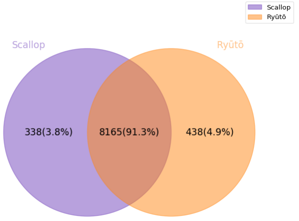

(m) ERR1138641 True Predictions

Figure S12: Venn diagram of reported true multi-exon transcripts of Scallop and Ryūtō.

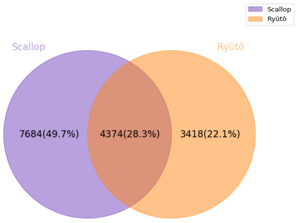

(a) T1 False Predictions

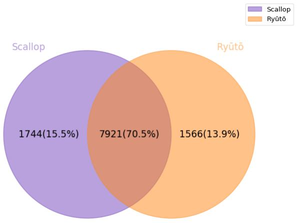

(b) EP False Predictions

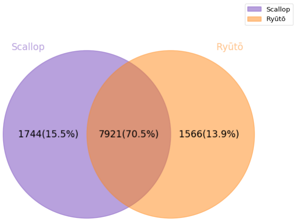

(c) ER False Predictions

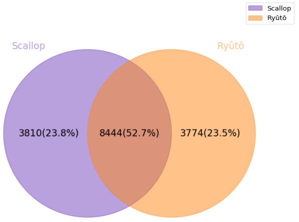

(d) SRR307911 False Predictions

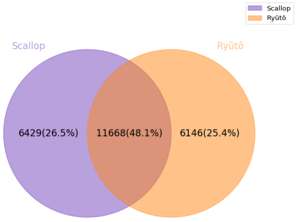

(e) SRR30791_1_2 False Predictions

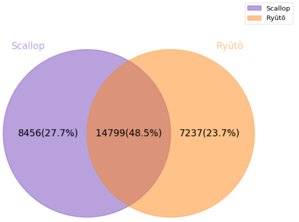

(f) SRR387661 False Predictions

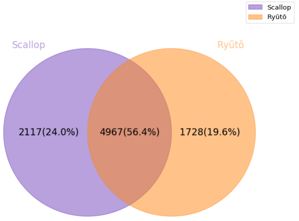

(g) SRR307903 False Predictions

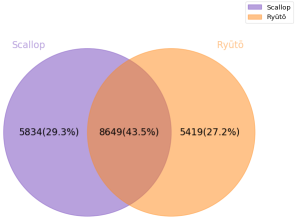

(h) SRR534319 False Predictions

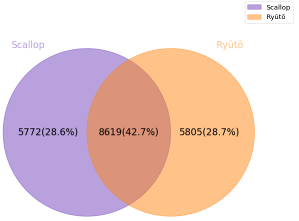

(i) SRR545695 False Predictions

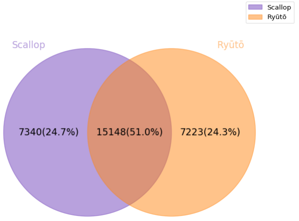

(j) SRR534307 False Predictions

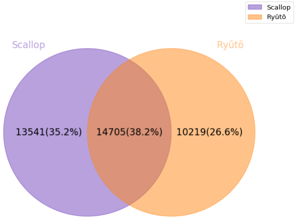

(k) SRR545723 False Predictions

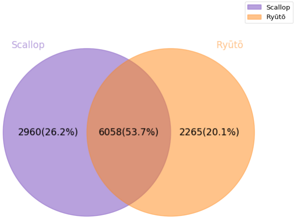

(l) SRR203276 False Predictions

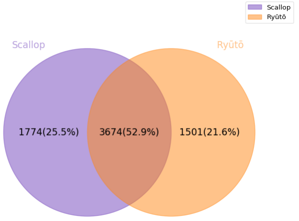

(m) ERR1138641 False Predictions

Figure S13: Venn diagram of reported false multi-exon transcripts of Scallop and Ryūtō.

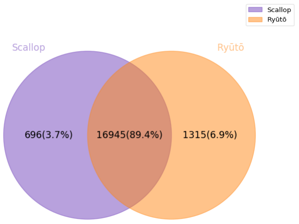

(a) T1 True Predictions

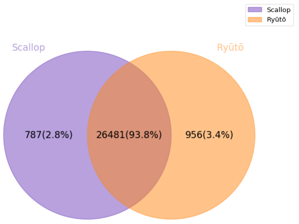

(b) EP True Predictions

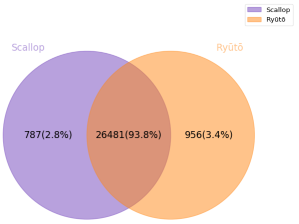

(c) ER True Predictions

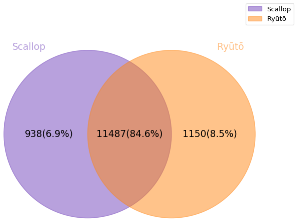

(d) SRR307911 True Predictions

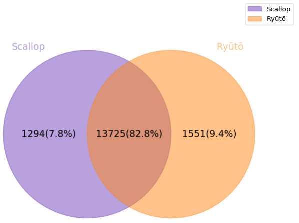

(e) SRR30791_1_2 True Predictions

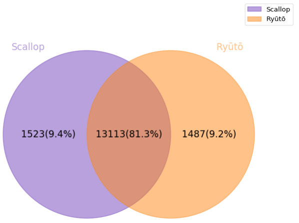

(f) SRR387661 True Predictions

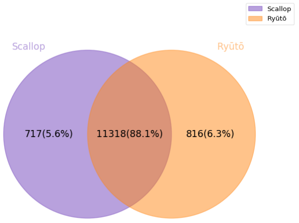

(g) SRR307903 True Predictions

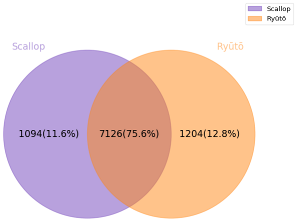

(h) SRR534319 True Predictions

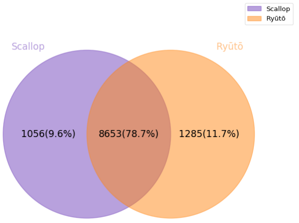

(i) SRR545695 True Predictions

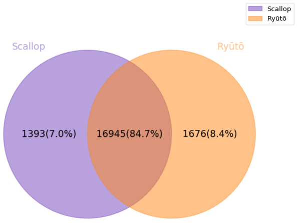

(j) SRR534307 True Predictions

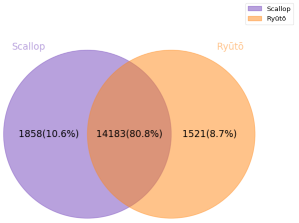

(k) SRR545723 True Predictions

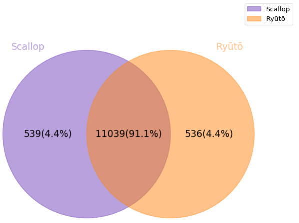

(l) SRR203276 True Predictions

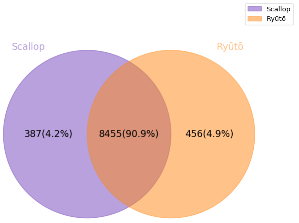

(m) ERR1138641 True Predictions

Figure S14: Venn diagram of reported true transcripts, including single exon transcripts, of Scallop and Ryūtō.

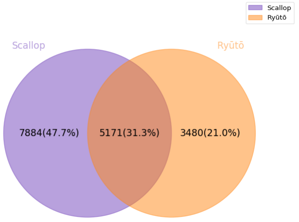

(a) T1 False Predictions

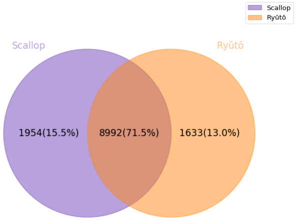

(b) EP False Predictions

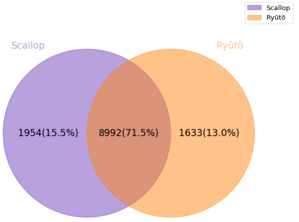

(c) ER False Predictions

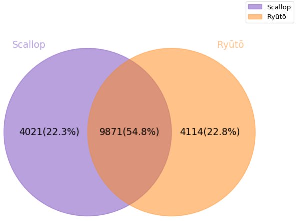

(d) SRR307911 False Predictions

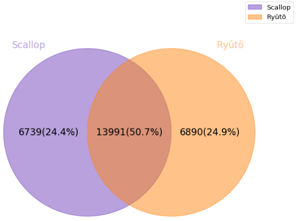

(e) SRR30791_1_2 False Predictions

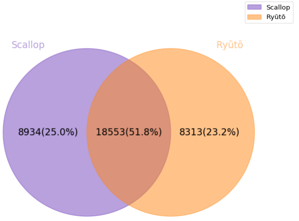

(f) SRR387661 False Predictions

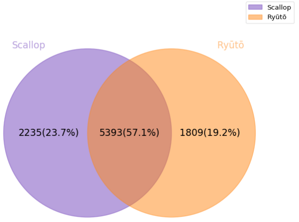

(g) SRR307903 False Predictions

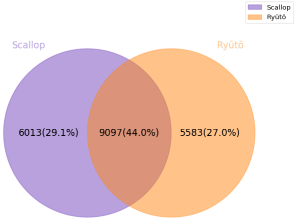

(h) SRR534319 False Predictions

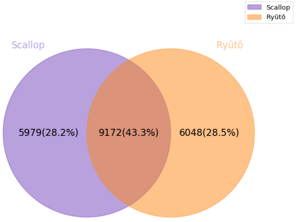

(i) SRR545695 False Predictions

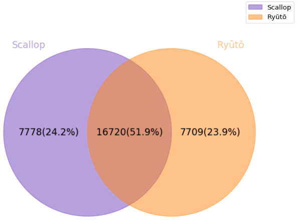

(j) SRR534307 False Predictions

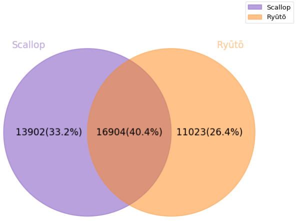

(k) SRR545723 False Predictions

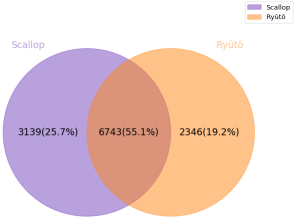

(l) SRR203276 False Predictions

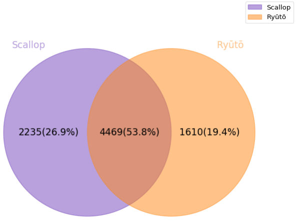

(m) ERR1138641 False Predictions

Figure S15: Venn diagram of reported false transcripts, including single exon transcripts, of Scallop and Ryūtō.

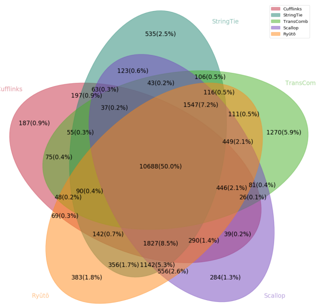

(a) T1 True Predictions

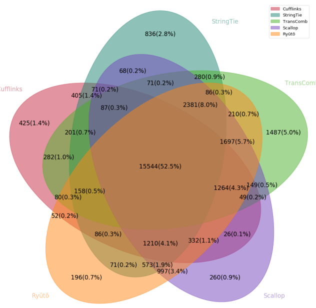

(b) EP True Predictions

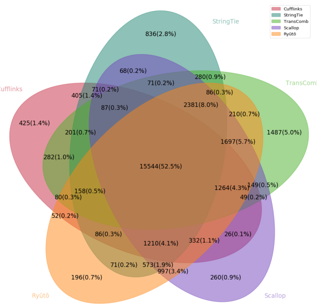

(c) ER True Predictions

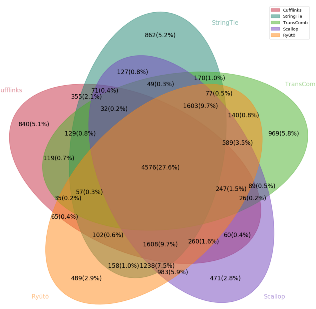

(d) SRR307911 True Predictions

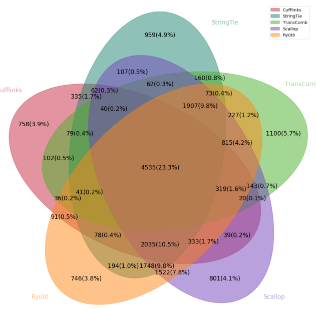

(e) SRR30791_1_2 True Predictions

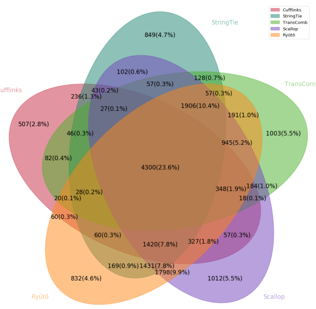

(f) SRR387661 True Predictions

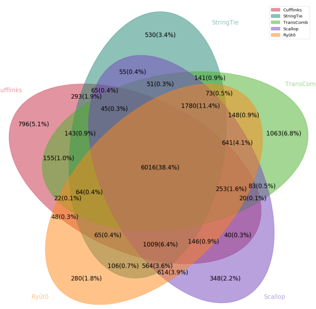

(g) SRR307903 True Predictions

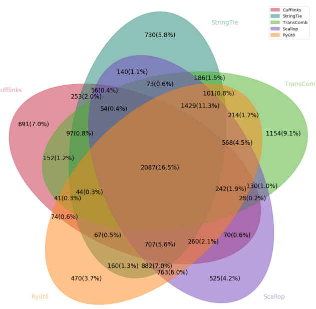

(h) SRR534319 True Predictions

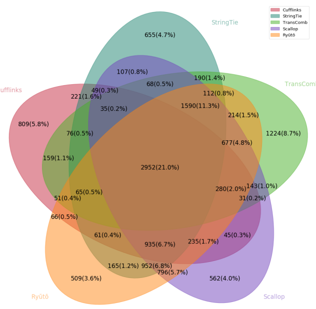

(i) SRR545695 True Predictions

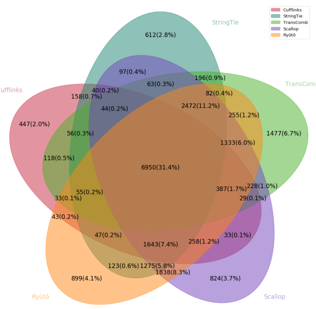

(j) SRR534307 True Predictions

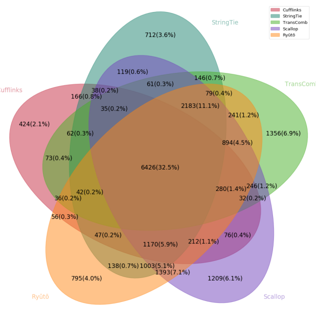

(k) SRR545723 True Predictions

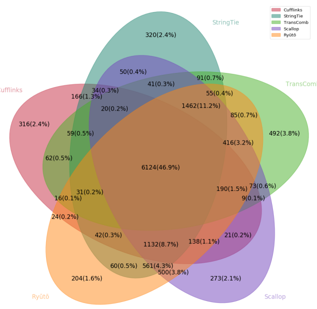

(l) SRR203276 True Predictions

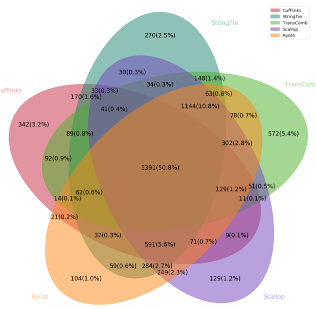

(m) ERR1138641 True Predictions

Figure S16: Venn diagram of reported true multi-exon transcripts of all tools.

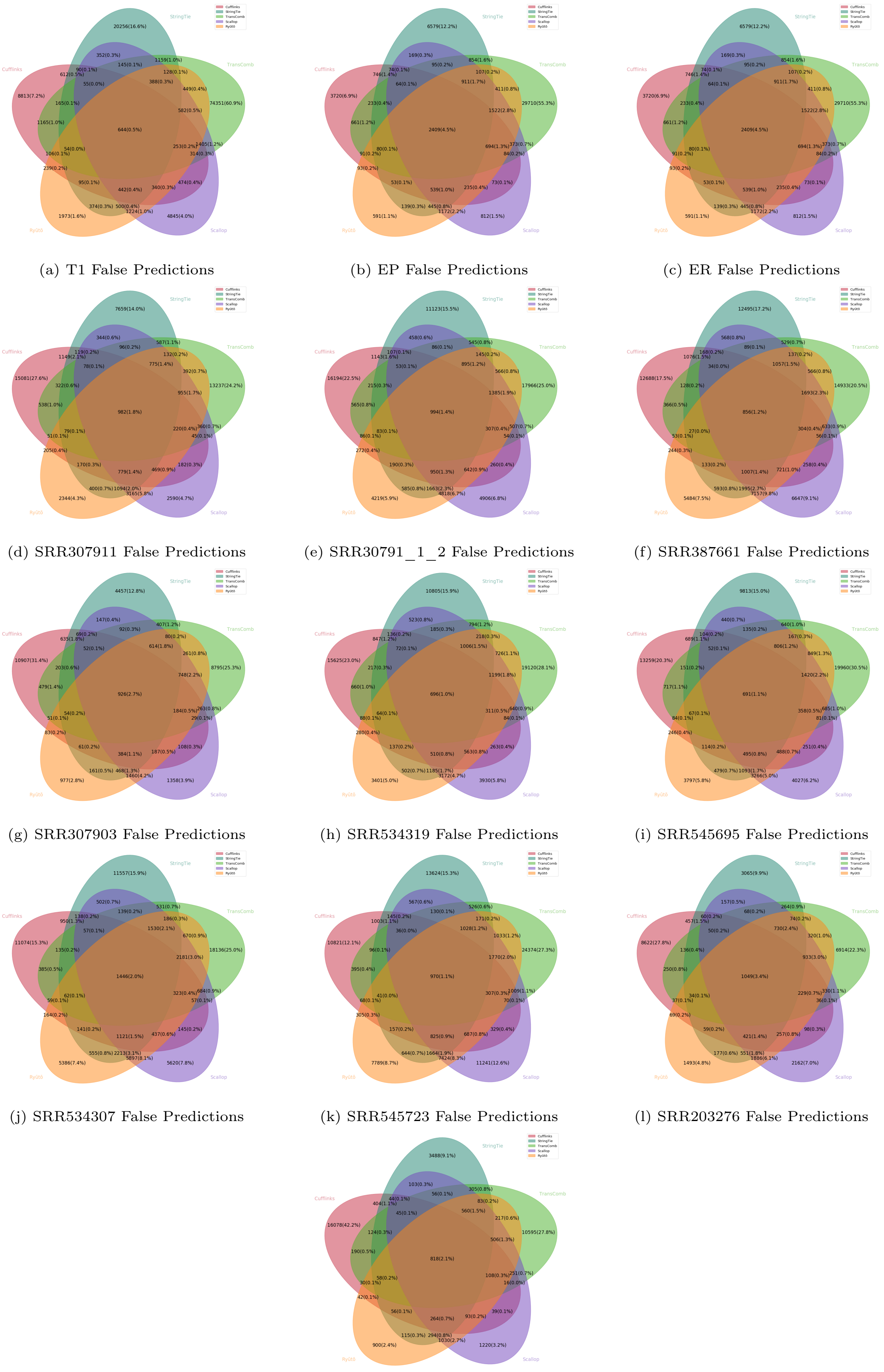

(a) T1 False Predictions

(b) EP False Predictions

(c) ER False Predictions

(d) SRR307911 False Predictions

(e) SRR30791_1_2 False Predictions

(f) SRR387661 False Predictions

(g) SRR307903 False Predictions

(h) SRR534319 False Predictions

(i) SRR545695 False Predictions

(j) SRR534307 False Predictions

(k) SRR545723 False Predictions

(l) SRR203276 False Predictions

(m) ERR1138641 False Predictions

Figure S17: Venn diagram of reported false multi-exon transcripts of all tools.

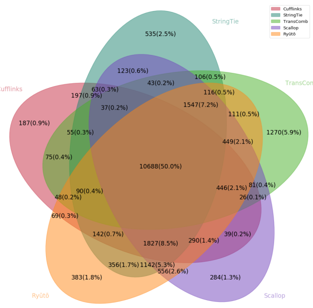

(a) T1 True Predictions

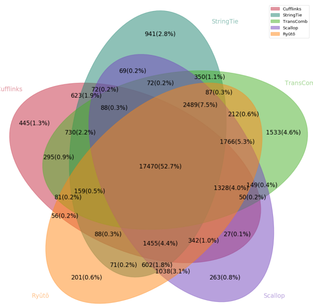

(b) EP True Predictions

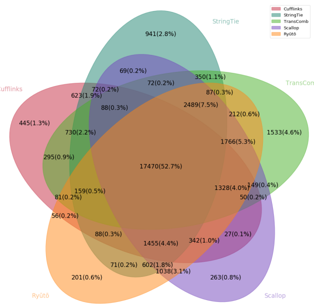

(c) ER True Predictions

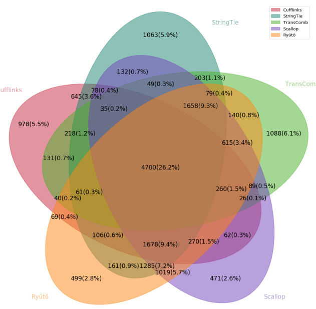

(d) SRR307911 True Predictions

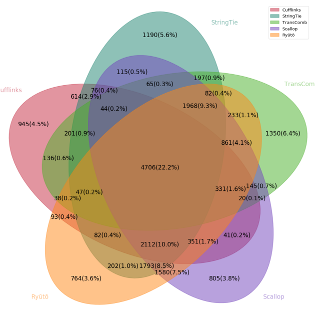

(e) SRR30791_1_2 True Predictions

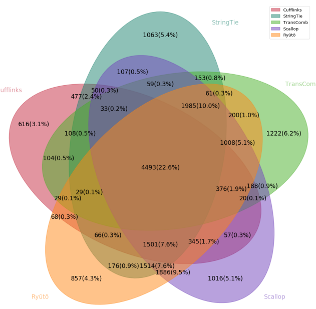

(f) SRR387661 True Predictions

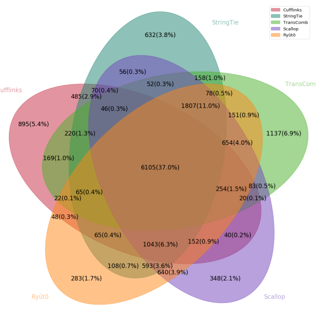

(g) SRR307903 True Predictions

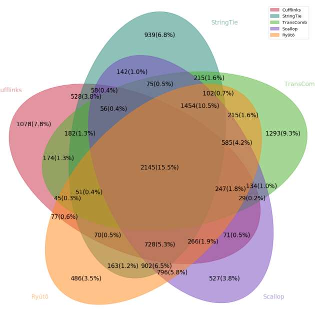

(h) SRR534319 True Predictions

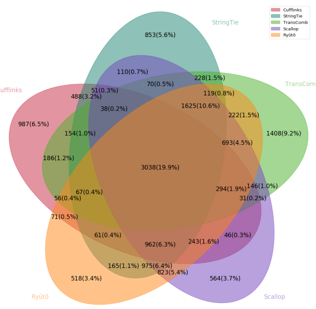

(i) SRR545695 True Predictions

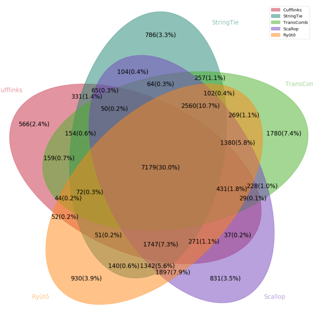

(j) SRR534307 True Predictions

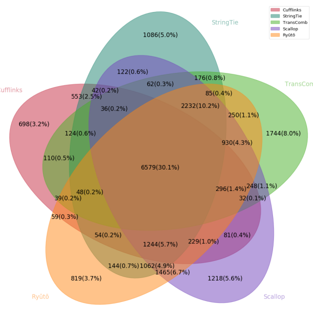

(k) SRR545723 True Predictions

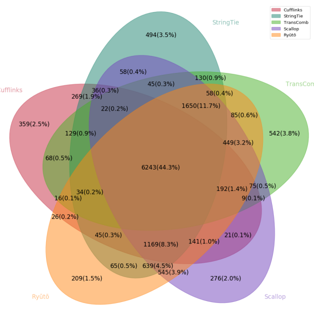

(l) SRR203276 True Predictions

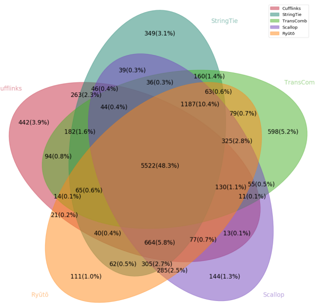

(m) ERR1138641 True Predictions

Figure S18: Venn diagram of reported true transcripts, including single exon transcripts, of all tools.

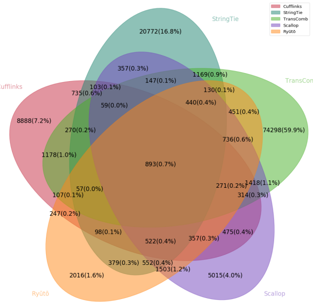

(a) T1 False Predictions

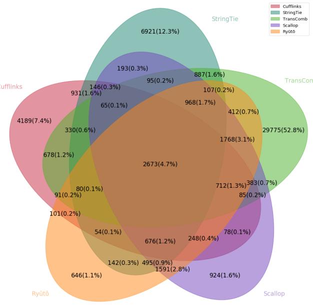

(b) EP False Predictions

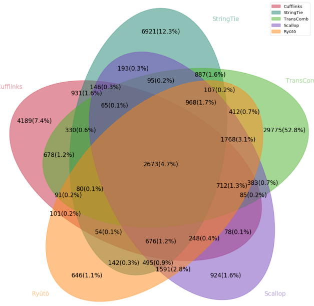

(c) ER False Predictions

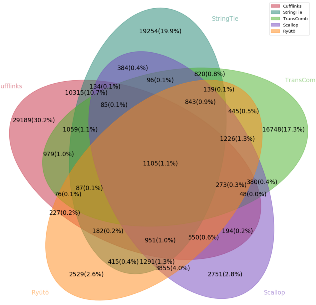

(d) SRR307911 False Predictions

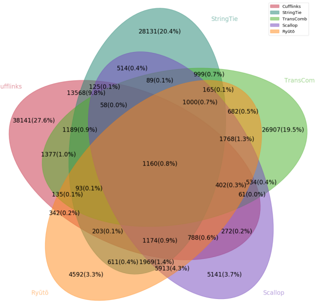

(e) SRR30791_1_2 False Predictions

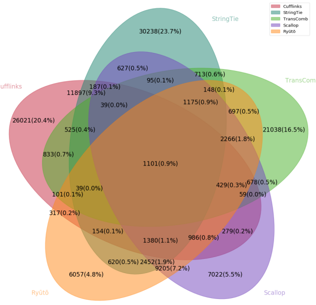

(f) SRR387661 False Predictions

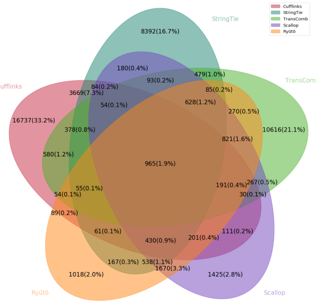

(g) SRR307903 False Predictions

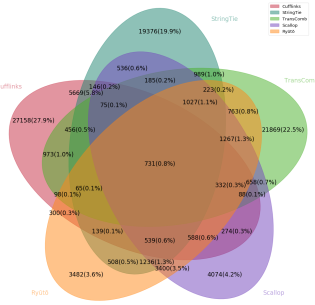

(h) SRR534319 False Predictions

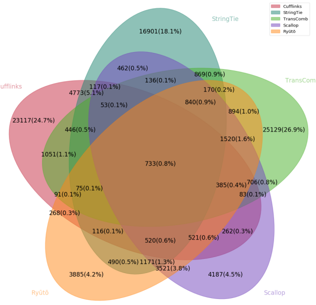

(i) SRR545695 False Predictions

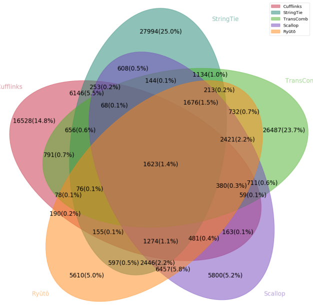

(j) SRR534307 False Predictions

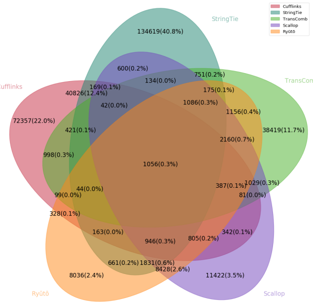

(k) SRR545723 False Predictions

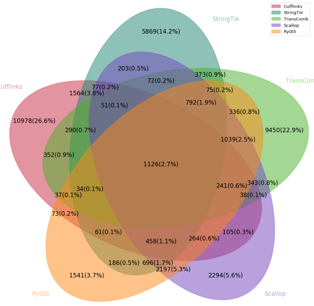

(l) SRR203276 False Predictions

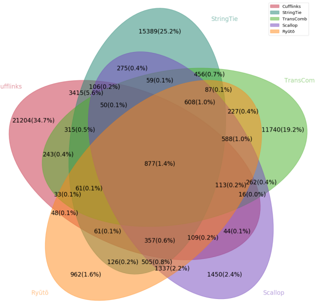

(m) ERR1138641 False Predictions

Figure S19: Venn diagram of reported false transcripts, including single exon transcripts, of all tools.

Additional Tables

Tool	***Wall Clock Time [s]***	***CPU use***	***RAM [MB]***
Cufflinks	388,794	457%	119700
StringTie	659.62	154%	547
Transcomb	4,393	87%	723
Scallop	660.28	98%	346
Ryūtō	958.6	357%	6910

Table S2: Running times, CPU use and memory use of benchmarked tools. We tested runtimes for ENSEMBL Realistic (ER) on a machine with an Intel(R) Xeon(R) CPU E7540 @ 2.00GHz and sufficient RAM. Ryūtō, Cufflinks and StringTie were run assigned 8 cores. Transcomb and Scallop do not offer this option and were run single threaded. Ryūtō took only slightly longer than StringTie despite the higher computational needs of Ryūtō, made up by more effective parallelization. The higher memory and time use of Ryūtō is explained by its internal infrastructure that is designed already for later addition of trans-splice and circularization events. Scallop can take advantage of its lower requirements for data-structures.

	Chromosome																				
	1	2	3	4	5	6	7	8	9	10	11	12	13	14	15	16	17	18	19	X	Y
ENSEMBL Perfect																					
ρ_C	**0.82**	*0.87*	**0.92**	**0.88**	**0.89**	**0.91**	**0.89**	**0.92**	**0.92**	**0.93**	**0.88**	**0.91**	**0.90**	**0.92**	**0.92**	*0.90*	**0.91**	**0.91**	**0.93**	*0.87*	*0.96*
ρ_S	**0.82**	0.86	0.88	*0.87*	*0.87*	0.89	0.87	*0.91*	0.89	*0.92*	**0.88**	**0.91**	0.88	*0.91*	*0.89*	**0.91**	0.88	0.88	*0.91*	0.85	0.94
ρ_T	*0.77*	0.80	0.84	0.82	0.83	0.85	0.84	0.83	0.84	0.77	*0.81*	0.83	0.78	0.82	0.79	0.78	0.81	0.82	0.80	0.75	0.94
ρ_R	**0.82**	**0.88**	*0.90*	**0.88**	**0.89**	*0.90*	*0.88*	**0.92**	*0.91*	0.82	*0.81*	*0.85*	*0.89*	**0.92**	**0.92**	**0.91**	*0.89*	*0.90*	*0.91*	**0.88**	**0.98**
ENSEMBL Realistic																					
ρ_C	**0.81**	*0.81*	**0.86**	**0.82**	**0.81**	**0.84**	**0.83**	**0.85**	**0.88**	**0.87**	**0.85**	**0.85**	**0.88**	**0.88**	**0.88**	0.83	**0.87**	**0.87**	**0.87**	**0.85**	0.86
ρ_S	*0.75*	0.78	*0.83*	*0.78*	*0.78*	0.81	*0.80*	*0.82*	0.84	*0.83*	*0.81*	**0.85**	0.84	*0.83*	*0.84*	*0.84*	0.83	0.79	0.83	0.80	*0.89*
ρ_T	0.72	0.77	0.81	0.76	0.76	0.78	0.79	0.79	0.75	0.68	0.79	*0.80*	0.80	0.76	0.76	0.77	0.82	0.78	0.81	0.71	**1.00**
ρ_R	*0.75*	**0.82**	**0.86**	**0.82**	**0.81**	*0.83*	**0.83**	**0.85**	*0.85*	0.74	0.74	0.77	*0.85*	**0.88**	*0.84*	**0.85**	*0.85*	*0.83*	*0.84*	*0.81*	*0.89*

Table S3: Spearman's rank correlation coefficients for Cufflinks ρ_C, StringTie ρ_S, Transcomb ρ_T and Ryūtō ρ_R for individual chromosomes of the simulated datasets ENSEMBL Perfect (EP) and ENSEMBL Realistic (ER). Ranks of predicted FPKM are correlated to true abundance ranks. Only the ranks of true predicted isoforms where considered. Cufflinks exhibits the highest accuracy, but also called the fewest transcripts, therefore gaining a slight advantage for this measure. StringTie and Ryūtō perform similarly well, with Ryūtō consistently in the advantage. The best results are highlighted bold, the second best italic.

Alignment	***Tool***	***1-10X***					***10-100X***					***>100X***				
		**TP**	**FP**	**Prec.**	**Rec.**	**F1**	**TP**	**FP**	**Prec.**	**Rec.**	**F1**	**TP**	**FP**	**Prec.**	**Rec.**	**F1**
TopHat	Cufflinks	-	-	-	-	-	-	-	-	-	-	14015	12110	53.65	32.60	40.55
	StringTie	-	-	-	-	-	-	-	-	-	-	16676	16851	49.74	38.79	43.58
	Transcomb 0	-	-	-	-	-	-	-	-	-	-	11783	29760	28.36	27.41	27.88
	Transcomb 4	-	-	-	-	-	-	-	-	-	-	11381	24773	31.48	26.47	28.76
	Scallop	-	-	-	-	-	-	-	-	-	-	16964	12566	57.45	39.46	46.78
	Ryūtō	-	-	-	-	-	-	-	-	-	-	17350	8906	66.08	40.35	**50.11**
STAR	Cufflinks	-	-	-	-	-	-	-	-	-	-	14271	13781	50.87	33.19	40.17
	StringTie	-	-	-	-	-	-	-	-	-	-	17025	25119	40.40	39.60	39.99
	Transcomb 0	-	-	-	-	-	-	-	-	-	-	13793	97760	12.36	32.08	17.85
	Transcomb 4	-	-	-	-	-	-	-	-	-	-	12717	83528	13.21	29.58	18.27
	Scallop	-	-	-	-	-	-	-	-	-	-	17619	11923	59.64	40.98	48.58
	Ryūtō	-	-	-	-	-	-	-	-	-	-	18245	7662	70.42	42.44	**52.96**
True	Cufflinks	-	-	-	-	-	-	-	-	-	-	18015	16007	52.95	41.90	46.78
	StringTie	-	-	-	-	-	-	-	-	-	-	19402	33690	36.54	45.13	40.38
	Transcomb 0	-	-	-	-	-	-	-	-	-	-	13663	59883	18.58	31.78	23.45
	Transcomb 4	-	-	-	-	-	-	-	-	-	-	13091	39232	25.02	30.45	27.47
	Scallop	-	-	-	-	-	-	-	-	-	-	20454	12257	62.53	47.57	54.04
	Ryūtō	-	-	-	-	-	-	-	-	-	-	21372	8571	71.38	49.71	**58.60**

Table S4: Total true predicted transcripts (TP), total false predicted transcripts (FP), recall, precision and F1 score for each tool broken down by the true abundance of transcripts on the theoretical simulated dataset T1.

Alignment	***Tool***	***1 Spliceform***			***2 Spliceforms***			***3 Spliceforms***			***4 Spliceforms***			***5 Spliceforms***		
		**TP**	**FP**	**F1**	**TP**	**FP**	**F1**	**TP**	**FP**	**F1**	**TP**	**FP**	**F1**	**TP**	**FP**	**F1**
TopHat	Cufflinks	878	203	**84.38**	2998	1831	55.38	3169	2823	42.28	3379	3578	35.65	3591	3675	32.26
	StringTie	894	283	82.13	3380	1953	59.66	3723	3586	45.66	4130	4898	39.28	4549	6131	35.43
	Transcomb 0	787	447	70.46	2497	3435	41.86	2640	5804	30.27	2790	8885	23.57	3069	11189	20.98
	Transcomb 4	787	346	73.79	2445	2926	43.01	2553	4915	31.01	2665	7445	24.11	2931	9141	21.65
	Scallop	894	230	84.18	3212	1878	57.94	3731	2762	48.16	4198	3720	42.16	4929	3976	41.24
	Ryūtō	894	228	84.26	3426	1295	**63.92**	3822	1925	**51.83**	4272	2605	**45.27**	4936	2853	**43.32**
STAR	Cufflinks	944	129	91.08	3101	2084	55.46	3222	3320	41.46	3347	4046	34.52	3657	4202	32.00
	StringTie	962	134	91.79	3557	2807	57.55	3808	5289	42.08	4172	7664	35.01	4526	9225	31.49
	Transcomb 0	942	4850	27.74	2861	17651	21.58	3019	21686	17.91	3314	25910	16.08	3657	27663	15.79
	Transcomb 4	870	4770	26.20	2647	15677	21.77	2765	18609	18.21	3073	21647	16.74	3362	22825	16.33
	Scallop	951	85	93.42	3371	1613	61.39	3855	2652	49.72	4431	3557	44.34	5011	4016	41.71
	Ryūtō	952	80	**93.70**	3658	953	**68.96**	4019	1709	**54.58**	4522	2222	**48.26**	5094	2698	**44.70**
True	Cufflinks	993	17	98.81	3888	2101	64.87	4056	4140	47.17	4398	4950	41.21	4680	4799	38.24
	StringTie	998	14	99.20	3889	3279	59.08	4325	7173	42.20	4805	10537	35.15	5385	12687	32.57
	Transcomb 0	998	20	98.91	2633	5859	36.34	2979	11580	25.29	3312	18686	19.48	3741	23738	17.61
	Transcomb 4	998	16	99.11	2547	4636	38.65	2853	8334	28.27	3139	11848	23.26	3554	14398	21.57
	Scallop	994	10	99.20	3889	1605	67.68	4460	2788	54.90	5146	3608	49.60	5965	4246	47.32
	Ryūtō	996	7	**99.45**	4227	1394	**72.76**	4657	1876	**59.96**	5366	2430	**54.22**	6126	2864	**51.07**

Table S5: Total true predicted transcripts (TP), total false predicted transcripts (FP) and F1 score for each tool broken down by number of spliceforms on each locus on the simulated dataset T1.

Alignment	***Tool***	***1-10X***					***10-100X***					***>100X***				
		**TP**	**FP**	**Prec.**	**Rec.**	**F1**	**TP**	**FP**	**Prec.**	**Rec.**	**F1**	**TP**	**FP**	**Prec.**	**Rec.**	**F1**
TopHat	Cufflinks	944	969	49.35	11.14	18.18	11385	6005	65.47	34.19	44.92	6808	3497	66.07	56.78	61.07
	StringTie	1246	1374	47.56	14.71	**22.47**	12859	7739	62.43	38.61	47.72	7216	4615	60.99	60.18	60.59
	Transcomb 0	1209	2705	30.89	14.27	19.52	9396	17108	35.45	28.22	31.42	4299	10133	29.79	35.85	32.54
	Transcomb 4	974	2550	27.64	11.50	16.24	9257	16001	36.65	27.80	31.62	4262	9522	30.92	35.55	33.07
	Scallop	1205	1055	53.32	14.22	22.46	14669	6586	69.01	44.05	53.78	7771	3761	67.39	64.81	66.07
	Ryūtō	1188	1070	52.61	14.02	22.14	14604	6374	69.62	43.85	**53.81**	7805	3702	67.83	65.10	**66.43**
STAR	Cufflinks	1061	952	52.71	12.52	20.24	12169	5544	68.70	36.54	47.71	7355	3245	69.39	61.34	65.12
	StringTie	1325	1251	51.44	15.64	**23.99**	13381	7555	63.91	40.18	49.34	7714	4403	63.66	64.34	64.00
	Transcomb 0	1399	4296	24.57	16.51	19.75	10657	27491	27.94	32.00	29.83	5409	16222	25.01	45.11	32.18
	Transcomb 4	1399	4296	24.57	16.51	19.75	10657	27491	27.94	32.00	29.83	5409	16222	25.01	45.11	32.18
	Scallop	1246	858	59.22	14.71	23.56	15606	5519	73.87	46.86	57.35	8245	3089	72.75	68.77	70.70
	Ryūtō	1271	880	59.09	15.00	23.93	15681	5369	74.49	47.09	**57.70**	8303	3052	73.12	69.25	**71.13**
True	Cufflinks	1291	742	63.50	15.24	24.58	13949	4441	75.85	41.89	53.97	8170	2681	75.29	68.14	71.54
	StringTie	1696	956	63.95	20.02	**30.49**	15263	5870	72.22	45.83	56.08	8285	3405	70.87	69.10	69.97
	Transcomb 0	1738	2340	42.62	20.51	27.70	11903	14849	44.49	35.74	39.64	5764	8569	40.21	48.07	43.79
	Transcomb 4	1363	2223	38.01	16.09	22.61	11743	13949	45.71	35.26	39.81	5706	8086	41.37	47.59	44.26
	Scallop	1452	678	68.17	17.14	27.39	17037	4583	78.80	51.16	**62.04**	8825	2588	77.32	73.60	75.42
	Ryūtō	1407	706	66.59	16.61	26.58	16999	4509	79.04	51.05	62.03	8892	2575	77.54	74.16	**75.82**

Table S6: Total true predicted transcripts (TP), total false predicted transcripts (FP), recall, precision and F1 score for each tool broken down by the true abundance of transcripts on the simulated dataset ENSEMBL Perfect.

Alignment	*Tool*	***1-10X***					***10-100X***					***>100X***				
		**TP**	**FP**	**Prec.**	**Rec.**	**F1**	**TP**	**FP**	**Prec.**	**Rec.**	**F1**	**TP**	**FP**	**Prec.**	**Rec.**	**F1**
TopHat	Cufflinks	1379	2217	38.35	9.00	14.58	12256	12196	50.12	34.45	40.83	2062	2248	47.84	56.26	51.71
	StringTie	1710	2621	39.48	11.17	**17.41**	13868	15524	47.18	38.98	42.69	2283	2723	45.61	62.29	52.66
	Transcomb 0	1747	4399	28.42	11.41	16.28	10042	26267	27.66	28.23	27.94	1281	4870	20.83	34.95	26.10
	Transcomb 4	1257	3918	24.29	8.21	12.27	9739	23512	29.29	27.38	28.30	1270	4416	22.34	34.65	27.16
	Scallop	1565	2604	37.54	10.22	16.06	14787	15238	49.25	41.56	45.08	2361	2931	44.61	64.42	52.72
	Ryūtō	1563	2110	42.55	10.21	16.46	15076	12392	54.89	42.38	**47.83**	2397	2346	50.54	65.40	**57.02**
STAR	Cufflinks	1437	2387	37.58	9.38	15.02	12547	13124	48.88	35.27	40.97	2133	2372	47.35	58.20	52.22
	StringTie	1784	2786	39.04	11.65	17.94	14125	16065	46.79	39.70	42.96	2304	2824	44.93	62.86	52.41
	Transcomb 0	1986	5251	27.44	12.97	17.61	10543	30865	25.46	29.64	27.39	1432	5918	19.48	39.07	26.00
	Transcomb 4	1316	4808	21.49	8.59	12.28	10132	28305	26.36	28.48	27.38	1400	5474	20.37	38.20	26.57
	Scallop	1625	2745	37.19	10.61	16.51	15218	15366	49.76	42.78	46.00	2312	3058	43.05	63.08	51.18
	Ryūtō	1731	2181	44.25	11.30	**18.01**	15723	12490	55.73	44.20	**49.30**	2394	2440	49.52	65.32	**56.34**
True	Cufflinks	1974	1786	52.50	12.89	20.70	15841	9560	62.36	44.53	51.96	2704	1677	61.72	73.78	67.21
	StringTie	2438	2039	54.46	15.92	**24.64**	17360	11955	59.22	48.80	53.51	2833	2089	57.56	77.30	65.98
	Transcomb 0	2676	3952	40.37	17.47	24.39	13492	23176	36.80	37.92	37.35	1951	4229	31.57	53.23	39.63
	Transcomb 4	1992	3596	35.65	13.01	19.06	13155	21166	38.33	36.98	37.64	1915	3885	33.02	52.25	40.46
	Scallop	2065	2206	48.35	13.48	21.09	17972	12698	58.60	50.52	54.26	2759	2448	52.99	75.28	62.20
	Ryūtō	2090	1583	56.90	13.65	22.01	18504	9151	66.91	52.01	**58.53**	2885	1724	62.59	78.72	**69.74**

Table S7: Total true predicted transcripts (TP), total false predicted transcripts (FP), recall, precision and F1 score for each tool broken down by the true abundance of transcripts on the simulated dataset ENSEMBL Realistic.

Alignment	*Tool*	***1-10X***					***10-100X***					***>100X***				
		**TP**	**FP**	**Prec.**	**Rec.**	**F1**	**TP**	**FP**	**Prec.**	**Rec.**	**F1**	**TP**	**FP**	**Prec.**	**Rec.**	**F1**
STAR	Cufflinks	3862	1172	76.72	25.22	37.96	20685	5970	77.60	58.14	66.48	3151	1091	74.28	85.98	**79.70**
	StringTie	9973	2196	81.95	65.12	72.57	27873	12377	69.25	78.35	73.52	2825	2209	56.12	77.08	64.95
	Ryūtō 10 & 9924	1974	83.41	64.80	72.94	28114	11206	71.50	79.03	75.07	2869	2124	57.46	78.28	66.27	
	Ryūtō 20	9919	1916	83.81	64.77	73.07	28082	10819	72.19	78.94	75.41	2867	2044	58.38	78.23	66.86
	Ryūtō 30	9915	1809	84.57	64.74	73.34	28058	10369	73.02	78.87	75.83	2865	1940	59.63	78.17	67.65
	Ryūtō 40	9908	1727	85.16	64.69	73.53	28013	9771	74.14	78.74	76.37	2860	1850	60.72	78.04	68.30
	Ryūtō 50	9911	1617	85.97	64.71	73.84	27966	9174	75.30	78.61	76.92	2852	1747	62.01	77.82	69.02
	Ryūtō 60	9899	1543	86.51	64.64	73.99	27881	8638	76.35	78.37	77.35	2835	1634	63.44	77.35	69.71
	Ryūtō 70	9898	1478	87.01	64.63	74.17	27851	8248	77.15	78.29	77.71	2831	1542	64.74	77.24	70.44
	Ryūtō 80	9893	1352	87.98	64.60	74.50	27796	7745	78.21	78.13	78.17	2819	1487	65.47	76.92	70.73
	Ryūtō 90	9893	1259	88.71	64.60	74.76	27753	7294	79.19	78.01	78.59	2798	1342	67.58	76.34	71.70
	Ryūtō 100	9846	402	96.08	64.29	**77.03**	26947	2406	91.80	75.74	**83.00**	2637	468	84.93	71.95	**77.90**

Table S8: Total true predicted transcripts (TP), total false predicted transcripts (FP), recall, precision and F1 score for each tool broken down by the true abundance of transcripts on the simulated dataset ENSEMBL Realistic. Tools were provided with a partly falsified annotation to guide assembly. Trust levels for Ryūtō are given.

Alignm. Tool	***Mode***	***1-10X***					***10-100X***					***>100X***				
		**TP**	**FP**	**Prec.**	**Rec.**	**F1**	**TP**	**FP**	**Prec.**	**Rec.**	**F1**	**TP**	**FP**	**Prec.**	**Rec.**	**F1**
STAR	*de novo*	845	2362	26.35	5.52	9.12	14064	13371	51.26	39.53	44.64	2285	2415	48.62	62.35	54.63
Stringtie	STAR+*de novo*	1809	2754	39.64	11.81	18.20	14474	15589	48.15	40.68	44.10	2338	2806	45.45	63.79	53.08
	STAR+*de novo* f	1586	2591	37.97	10.36	16.27	14407	14798	49.33	40.50	44.48	2338	2648	46.89	63.79	54.05
STAR	*de novo*	821	2297	26.33	5.36	8.91	14805	13195	52.88	41.62	46.57	2335	2560	47.70	63.71	54.56
Scallop	STAR+*de novo*	1879	3061	38.04	12.27	18.55	15675	17208	47.67	44.06	45.79	2325	3406	40.57	63.44	49.49
	STAR+*de novo* f	1609	2703	37.31	10.51	16.40	15488	15121	50.60	43.53	46.80	2320	2977	43.80	63.30	51.77
STAR	*de novo*	878	2096	29.52	5.73	9.60	15307	11990	56.08	43.03	48.69	2380	2349	50.33	64.94	56.71
Ryūtō	STAR+*de novo*	1928	2373	44.83	12.59	19.66	16090	13669	54.07	45.23	49.25	2396	2632	47.65	65.38	55.12
	STAR+*de novo* f	1715	2165	44.20	11.20	17.87	15955	12499	56.07	44.85	49.84	2396	2419	49.76	65.38	56.51
HISAT	*de novo*	897	1966	31.33	5.86	9.87	15247	11512	56.98	42.86	48.92	2486	2097	54.24	67.83	60.28
StringTie	STAR+*de novo*	1832	2472	42.57	11.96	18.68	15247	14366	51.49	42.86	46.78	2485	2553	49.33	67.80	57.11
	STAR+*de novo* f	1603	2325	40.81	10.47	16.66	15186	13601	52.75	42.69	47.19	2485	2405	50.82	67.80	58.09
HISAT	*de novo*	803	2060	28.05	5.24	8.83	15791	11765	57.31	44.39	50.03	2579	2237	53.55	70.37	60.82
Scallop	STAR+*de novo*	1907	2828	40.27	12.45	19.02	16548	16039	50.78	46.51	48.55	2508	3144	44.37	68.43	53.84
	STAR+*de novo* f	1637	2440	40.15	10.69	16.88	16322	13997	53.83	45.88	49.54	2501	2721	47.89	68.24	56.28
HISAT	*de novo*	898	1864	32.51	5.86	9.94	16232	10871	59.89	45.63	51.79	2593	2050	55.85	70.75	62.42
Ryūtō	STAR+*de novo*	1972	2170	47.61	12.88	20.27	16943	12583	57.38	47.62	52.05	2571	2419	51.52	70.15	59.41
	STAR+*de novo* f	1749	1952	47.26	11.42	18.40	16801	11407	59.56	47.23	52.68	2571	2183	54.08	70.15	61.08

Table S9: Total true predicted transcripts (TP), total false predicted transcripts (FP), recall, precision and F1 score for Ryūtō and StringTie. Paired-end reads of the simulated dataset ENSEMBL Realistic were aligned using STAR. Assembled *de novo* super-reads of the same data were aligned with STAR or HISAT. Results for only the *de novo* alignment, and a merged dataset of paired-end and *de novo* are given, run at standard settings, or with higher filters (f). Results are broken down according to abundance of the true transcripts.

Tool	***1-10X***					***10-100X***					***>100X***				
	**TP**	**FP**	**Prec.**	**Rec.**	**F1**	**TP**	**FP**	**Prec.**	**Rec.**	**F1**	**TP**	**FP**	**Prec.**	**Rec.**	**F1**
Ryūtō alt.	1685	2158	43.85	11.00	17.59	15578	12398	55.68	43.79	49.02	2375	2421	49.52	64.80	56.14
Ryūtō norm.	1731	2181	44.25	11.30	**18.01**	15723	12490	55.73	44.20	**49.30**	2394	2440	49.52	65.32	**56.34**

Table S10: Total true predicted transcripts (TP), total false predicted transcripts (FP), recall, precision and F1 score for the standard definition (norm.) and the alternative definition (alt.) of Ryūtō broken down by the true abundance of transcripts on the simulated dataset ENSEMBL Realistic.

Dataset	***Tool***	**TP**	**FP**	**Prec.**	**Rec.**	**F1**
SRR307911	Cufflinks	8649	20418	29.76	5.04	8.62
	StringTie	11324	14694	43.52	6.60	11.46
	Transcomb	8985	18814	32.32	5.23	9.01
	Scallop	12169	12167	50.00	7.09	12.42
	Ryūtō	12368	12127	50.49	7.21	**12.61**
SRR30791_1_2	Cufflinks	8973	22068	28.91	5.23	8.85
	StringTie	12532	19160	39.54	7.30	12.33
	Transcomb	9751	24412	28.54	5.68	9.48
	Scallop	14659	17986	44.90	8.54	14.35
	Ryūtō	14865	17708	45.64	8.66	**14.56**
SRR387661	Cufflinks	7640	18081	29.70	4.45	7.74
	StringTie	10985	20810	34.55	6.40	10.80
	Transcomb	9439	21421	30.59	5.50	9.32
	Scallop	14151	23155	37.93	8.24	**13.54**
	Ryūtō	14057	21940	39.05	8.19	**13.54**
SRR307903	Cufflinks	9246	14361	39.17	5.39	9.47
	StringTie	11143	8732	56.07	6.49	11.64
	Transcomb	10837	13162	45.16	6.31	11.08
	Scallop	11903	6992	63.00	6.93	12.49
	Ryūtō	12001	6601	64.51	6.99	**12.62**
SRR534319	Cufflinks	5159	20526	20.09	3.01	5.23
	StringTie	7124	17850	28.53	4.15	7.25
	Transcomb	6638	26062	20.30	3.87	6.50
	Scallop	8087	14435	35.91	4.71	8.33
	Ryūtō	8176	14023	36.83	4.76	**8.44**
SRR545695	Cufflinks	6126	17809	25.59	3.57	6.26
	StringTie	8321	15876	34.39	4.85	8.50
	Transcomb	7944	26818	22.85	4.63	7.70
	Scallop	9573	14316	40.07	5.58	9.79
	Ryūtō	9769	14350	40.50	5.69	**9.98**
SRR534307	Cufflinks	10417	16641	38.50	6.07	10.48
	StringTie	14097	21177	39.96	8.21	13.62
	Transcomb	13949	26506	34.48	8.13	13.15
	Scallop	17747	22364	44.24	10.34	16.76
	Ryūtō	17927	22246	44.62	10.44	**16.93**
SRR545723	Cufflinks	9248	16244	36.28	5.39	9.38
	StringTie	12599	21546	36.90	7.34	12.24
	Transcomb	12357	31984	27.87	7.20	11.44
	Scallop	15618	28132	35.70	9.10	14.50
	Ryūtō	15221	24817	38.02	8.87	14.38
	Ryūtō –keep-introns	15466	26193	37.13	9.01	14.50
	Ryūtō -c 3.8 –keep-introns	15629	27014	36.65	9.10	**14.59**
SRR203276	Cufflinks	8483	11770	41.89	10.30	16.53
	StringTie	10388	7232	58.96	12.61	20.78
	Transcomb	9333	11372	45.08	11.33	18.11
	Scallop	11191	8899	55.70	13.59	21.85
	Ryūtō	11183	8206	57.68	13.58	**21.98**
ERR1138641	Cufflinks	7185	18330	28.16	8.72	13.32
	StringTie	8549	6715	56.01	10.38	17.51
	Transcomb	8310	13876	37.46	10.09	15.90
	Scallop	8605	5346	61.68	10.45	17.87
	Ryūtō	8703	5075	63.17	10.57	**18.11**

Table S11: Total true predicted transcripts (TP), total false predicted transcripts (FP), recall, precision and F1 score for each tool broken down by the true abundance of transcripts on realistic data, looking only at multi-exon transcripts.

Dataset	***Tool***	**TP**	**FP**	**Prec.**	**Rec.**	**F1**
SRR307911	Cufflinks	9440	45388	17.22	4.80	7.51
	StringTie	12073	33055	26.75	6.14	9.99
	Transcomb	9683	25267	27.71	4.93	8.37
	Scallop	12518	13800	47.56	6.37	11.24
	Ryūtō	12717	13804	47.95	6.47	**11.40**
SRR30791_1_2	Cufflinks	9931	59018	14.40	5.05	7.48
	StringTie	13407	45185	22.88	6.82	10.51
	Transcomb	10782	38688	21.80	5.49	8.77
	Scallop	15141	20609	42.35	7.70	13.04
	Ryūtō	15355	20632	42.67	7.81	**13.21**
SRR387661	Cufflinks	8454	44289	16.03	4.30	6.78
	StringTie	11811	45483	20.61	6.01	9.31
	Transcomb	10404	31043	25.10	5.29	8.74
	Scallop	14751	27373	35.02	7.51	**12.36**
	Ryūtō	14671	26510	35.63	7.47	12.34
SRR307903	Cufflinks	9779	23625	29.27	4.98	8.51
	StringTie	11687	15503	42.98	5.95	10.45
	Transcomb	11280	15684	41.83	5.74	10.09
	Scallop	12133	7531	61.70	6.17	11.22
	Ryūtō	12220	7082	63.31	6.22	**11.32**
SRR534319	Cufflinks	5852	37594	13.47	2.98	4.88
	StringTie	7713	29044	20.98	3.92	6.61
	Transcomb	7171	30034	19.27	3.65	6.14
	Scallop	8271	15060	35.45	4.21	7.52
	Ryūtō	8368	14603	36.43	4.26	**7.62**
SRR545695	Cufflinks	6850	32553	17.38	3.49	5.81
	StringTie	8933	25517	25.93	4.55	7.74
	Transcomb	8560	33387	20.41	4.36	7.18
	Scallop	9789	15072	39.37	4.98	8.84
	Ryūtō	10009	15099	39.86	5.09	**9.03**
SRR534307	Cufflinks	11336	28847	28.21	5.77	9.58
	StringTie	15120	44191	25.49	7.69	11.82
	Transcomb	15040	37802	28.46	7.65	12.06
	Scallop	18489	24348	43.16	9.41	15.45
	Ryūtō	18683	24141	43.63	9.51	**15.61**
SRR545723	Cufflinks	10328	119023	7.98	5.26	6.34
	StringTie	13661	166094	7.60	6.95	7.26
	Transcomb	13492	51070	20.90	6.87	10.34
	Scallop	16178	30670	34.53	8.23	13.30
	Ryūtō	15770	27724	36.26	8.02	13.14
	Ryūtō –keep-introns	16015	29119	35.48	8.15	13.25
	Ryūtō -c 3.8 –keep-introns	16175	29891	35.11	8.23	**13.34**
SRR203276	Cufflinks	8886	15648	36.22	9.31	14.81
	StringTie	11180	11281	49.78	11.71	18.96
	Transcomb	10035	14707	40.56	10.51	16.69
	Scallop	11702	9759	54.53	12.25	20.01
	Ryūtō	11676	8941	56.63	12.23	**20.11**
ERR1138641	Cufflinks	7714	26970	22.24	8.08	11.85
	StringTie	9091	22266	28.99	9.52	14.33
	Transcomb	8783	15991	35.45	9.20	14.61
	Scallop	8948	6599	57.55	9.37	16.12
	Ryūtō	8996	5926	60.29	9.42	**16.29**

Table S12: Total true predicted transcripts (TP), total false predicted transcripts (FP), recall, precision and F1 score for each tool broken down by the true abundance of transcripts on realistic data, looking at all transcripts including single exon transcripts.

References

Boost C++ Libraries. URL `http://www.boost.org/`.

CLP C++ Libraries. URL `https://projects.coin-or.org/Clp`.

HTSlib C++ Library. URL `http://www.htslib.org/`.

LEMON C++ Libraries. URL `http://lemon.cs.elte.hu/trac/lemon`.

Alexander Dobin, Carrie A Davis, Felix Schlesinger, Jorg Drenkow, Chris Zaleski, Sonali Jha, Philippe Batut, Mark Chaisson, and Thomas R Gingeras. STAR: ultrafast universal RNA-seq aligner. *Bioinformatics*, 29(1):15–21, 2013.

Katharina E Hayer, Angel Pizarro, Nicholas F Lahens, John B Hogenesch, and Gregory R Grant. Benchmark analysis of algorithms for determining and quantifying full-length mRNA splice forms from RNA-seq data. *Bioinformatics*, 31(24):3938–3945, 2015.

Daehwan Kim, Geo Pertea, Cole Trapnell, Harold Pimentel, Ryan Kelley, and Steven L Salzberg. TopHat2: accurate alignment of transcriptomes in the presence of insertions, deletions and gene fusions. *Genome biology*, 14(4):R36, 2013.

Daehwan Kim, Ben Langmead, and Steven L Salzberg. HISAT: a fast spliced aligner with low memory requirements. *Nature methods*, 12(4):357–360, 2015.
